# Synthesis and *in-vitro* anti-proliferative with antimicrobial activity of new coumarin containing heterocycles hybrids

**DOI:** 10.1038/s41598-023-50170-9

**Published:** 2023-12-21

**Authors:** Eman Abdelaziz, Nehal M. El-Deeb, Mervat F. Zayed, Asmaa Mohammed Hasanein, Ibrahim El-T. El Sayed, Elshaymaa I. Elmongy, Elbadawy A. Kamoun

**Affiliations:** 1 Department of Chemistry, Faculty of Science, Menoufia University, Menoufia, Egypt; 2 Biopharmaceutical Products Research Department, Genetic Engineering and Biotechnology Research Institute (GEBRI) City of Scientific Research and Technological Applications (SRTA-City) Alexandria, New Borg El-Arab City, 21934 Egypt; 3 Department of Pharmaceutical Chemistry, Faculty of Pharmacy, Helwan University, Ain Helwan, P.O. Box 11795, Cairo, Egypt; 4 Polymeric Materials Research Department, Advanced Technology and New Materials Research Institute (ATNMRI), City of Scientific Research and Technological Applications (SRTA-City, Alexandria, New Borg El-Arab City, 21934 Egypt; 5 Nanotechnology Research Center (NTRC), The British University in Egypt (BUE), El-Sherouk City, Cairo, Egypt

**Keywords:** Biophysics, Cancer, Medical research, Chemistry

## Abstract

A series of new coumarin-*N-*heterocyclic hybrids, coumarin-quinolines **7a–e,** coumarin-acridines **10b,c** and coumarin-neocryptolepines **13b,c** were synthesized and evaluated for their anticancer and antimicrobial activities. The structures of all synthesized hybrids were confirmed by FT-IR, ^1^H-NMR, ^13^C-NMR, and MS spectrometry. The anti-proliferative activity of hybrids **7a–e, 10c** and **13c** were bio-evaluated using MTT-assay against colon (*CaCo-2*), lung (*A549*), breast (*MDA-MB-231*), and hepatocellular carcinoma (*HepG-2*) human cancer cell lines using doxorubicin as a reference drug. The results demonstrated that, all hybrids displayed moderate to good anti-proliferative activity against the cell lines. The most active hybrids were **7a–d** and **10c** against *CaCo-2* cancer cell line with IC_50_: 57.1, 52.78, 57.29, 51.95 and 56.74 µM, and selectivity index 1.38, 1.76, 2.6, 1.96 and 0.77; respectively. While, **7a**,**d** were potent against A*549* cancer cell line with IC_50_: 51.72, 54.8 µM and selectivity index 1.5, 0.67; respectively. Moreover, **7c** showed the most potency against *MDA-MB-231* cancer cell line with IC_50_: 50.96 µM and selectivity index 2.20. Interestingly, docking results revealed that binding energy of the current compounds showed marked affinity values ranging from -6.54 to -5.56 kcal with interactions with the reported key amino acid SER 79. Furthermore, the antimicrobial activity of the synthesized hybrids **7a–e, 10b,c, 13b and 13c** were evaluated against Gram‐positive and Gram‐negative bacterial and fungal strains. The hybrids **10b, 13b, 10c,** and **13c** exhibited broad-spectrum antibacterial activity against *E.coli, S. mutans, and S. aureus* with MIC from 3.2 to 66 µM, this hybrids also displayed antifungal activity against *C. albicans* with MIC values ranging from 0.0011 to 29.5 µM. In-silico investigation of the pharmacokinetic properties indicated that tested hybrids had high GI absorption, low Blood Brain Barrier (BBB) permeability in addition to cell membrane penetrability.

## Introduction

In recent years, the number of people suffering from cancer and multi-resistant infections has increased, such that both diseases are already seen as current and future major causes of death. Moreover, chronic infections are one of the main causes of cancer, due to the instability in the immune system that allows cancer cells to proliferate. Likewise, the physical debility associated with cancer or with anticancer therapy itself often paves the way for opportunistic infections. Though advances in cancer therapy and diagnosis have considerably improved life expectancy^1^, the overall survival rate of patients remains poor^2^. Disseminated cancer at the time of diagnosis and acquisition of tumor resistance are two main reasons. The growing knowledge of the biochemical pathways involved in a disease process increases the possibility to develop new approaches to treat this disease^3,4^. However, there are two main challenges to using chemotherapy successfully in cancer treatment are tumor cell resistance and the need for tumor cell specificity to prevent toxicity to normal tissues. Consequently, targeting pathways that selectively inhibit cancer cells growth and metastasis may provide successful anticancer therapy^5^. Due to the possibility of life‐threatening infections developing during anticancer treatment for cancer patients which are associated with prolonged hospital stay and poor quality of life^6^, many researchers are working to synthesize new drugs that have both anticancer and antimicrobial activity^7–9^.

**Figure 1:**
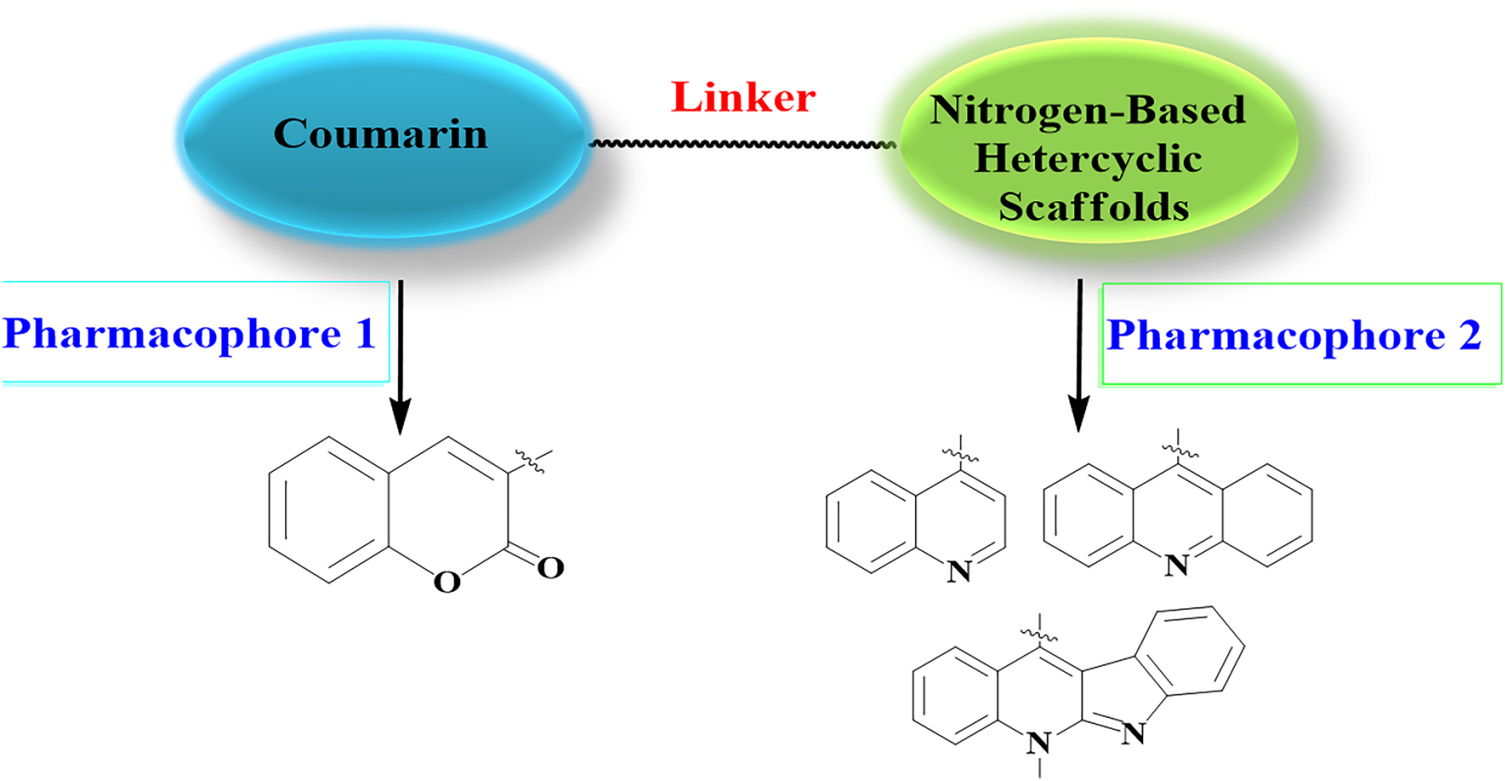
Design of coumarin *N-*heterocyclic hybrids as potent antiproliferative and antimicrobial agents.

During the last few years molecular hybridization became a powerful tool in drug design and discovery offering an attractive approach to obtain better drugs for the treatment of a large variety of human diseases including cancer and microbial illness. One of the methods used for the construction of hybrid molecules combines two or more drug pharmacophores in a single multi-functional molecule using a linker chain. The main goals of this pharmacophore merging approach consist in the interaction of the resulting molecule with dual or multiple targets, amplifying the biological activity and specificity, reducing the known side effects associated with each hybrid part, reducing the drug-drug interactions when compared with conventional classic drugs^10–12^. In this context, coumarin constitutes a unique motif for the construction of various classes of biologically active analogues with wide variety of pharmacological activities^13^, such as antidepressants^14^, antimicrobials^15–18^, antioxidants^19–21^, anti-inflammatories^20,22^, antinociceptives^23^, antitumors^24–26^, antiasthmatics^27^, antivirals^28^, antifungal^29^ and anti-coagulant activities.^30,31^ Previous studies showed that the hybridization of coumarin at C-4 and C-3 positions with heterocycles have synergistic effect on biological activity.^32–38^ Possible modifications of the coumarin core structures with respect to improving their anticancer and antimicrobial activities would be the installation of different side chains at position 3 and the identity of the spacer influences the flexibility of the structure and hence weakens or enforces interaction with certain targeted biomolecules. Such substitution patterns would affect and improve biological activity. Based on the forementioned information, it might be assumed that the installation of biologically effective *N-*heterocyclic scaffolds with natural origin such as quinoline^39^, acridine^40^ and neocryptolepine^41^ motifs into the coumarin core structure would improve the coumarin pharmacological profile as anticancer and antimicrobial agent with synergistic effects when compared with each moiety separately as depicted in Fig. 1. Herein, a series of new coumarin based heterocyclic hybrids are synthesized, and their anti-proliferative and antimicrobial activity are evaluated. In addition, a molecular docking study was executed to confirm binding of hybrids with the target protein structures.

## Materials and methods

### Materials

4,7-Dichloroquinoline 97%, ethylenediamine 99%, hydrazine hydrate 98%, 4,4'-methylenedianiline 97%, triethylamine 99%, coumarin-*3*-carboxylic acid 99%, were purchased from Sigma Aldrich, MO, USA. Also, reagents as 1,3-Diaminopropane 99%, 1,4-Phenylenediamine, thionyl chloride 97% and solvents as ethanol 98%, dichloromethane 98%, and petroleum ether with 60–80 °C, were supplied from LOBA Chemie, Mumbai, India and used without any further purification. The prepared starting materials such as 9-chloroacridne and 11-chloroneocryptolepine and their corresponding free amines were prepared as reported^42,43^.

#### Cell lines and microbial strains

*Cell lines*; WISH cell line (Human amnion-derived normal epithelial cells, CCL-25), *MDA-MB-231* cell line (Human, mammary gland/breast adenocarcinoma, HTB-26), *CaCo-2* cell line (Human Colorectal adenocarcinoma, epithelial cells, HTB-37), *A549* cell line (Human Lung adenocarcinoma, epithelial cells, CCL-185) cell lines and *HepG-2* (liver hepatocellular carcinoma, HEPG2, HB-8065) were obtained from ATCC, originally with license and serial numbers as mentioned.

*Microbial strains: Escherichia coli*, *pseudomonas aeruginosa, Staphylococcus aureus, Streptococcus mutans* and *Candida albicans* were collected from the microbial culture collection of Biopharmaceutical Products Research Department, Genetic Engineering and Biotechnology Research Institute, SRTA-City, New Borg El-Arab city, Alexandria, Egypt.

#### Molecular docking

Molecular docking studies were effectuated using and visualized on *Biovia DS*-2021 online free software to examine its affinity to topoisomerase II(Topo-II). Topo-II protein (pdb: 3FOE) was downloaded from protein data bank^44^. Protein preparation was done by deleting water molecules, selecting the co-crystalized ligand to be used as a current selection for defining the active site of the Top II and create binding site sphere attributes which was then extended to cover the whole protein. The ligand was then deleted, polar hydrogen was added, and files are saved as optimized protein. Ligand optimization and docking was performed by selecting dock ligand in lib-dock to prepare the ligand automatically in the program using the input site sphere attributes which was created upon protein optimization and minimize energy for the macromolecules before docking. The selected minimization force field was CHARMm.

### Instrumental characterization

^**1**^**H-NMR** and ^**13**^**C-NMR** spectroscopic analyses were carried out with Bruker Germany; 400 and 100 MHz; respectively. Reports of chemical shifts were made in parts per million (ppm) as it relates to the respective solvent (DMSO-d_6_). Also, **FT-IR** spectra were performed with Alpha, Bruker Germany at faculty of Science, Zagazig University. Thermo-Scientific GCMS model ISQ, USA, was used for the mass-spectra, which were conducted on the direct inlet part; at the regional center for Mycology and Biotechnology (RCMB). Exploring the characteristic fragmentation using Electron Impact mode and expected molecular weight at 70 eV. Melting point was recorded using scientific melting point apparatus without correction.

### General synthesis of coumarin- N-heterocyclic hybrids *7a–e, 10b,c and 13b,c*.

Coumarin-*3*-carbonyl chloride **3** (0.30 g, 1.27 mmol), appropriate amines **6a–e, 9b,c** and **12b,c** (1.27 mmol) dissolved in CH_2_Cl_2_ (2 mL), triethylamine (0.39 g, 3.81 mmol) was added dropwise with stirring at room temperature (25˚C). Progress of the reaction was observed by thin layer chromatography (TLC) till starting materials were consumed (12 h.). The reaction mixture was poured into ice/water, and extracted three times with CH_2_Cl_2,_ the organic layer was collected, dried followed by removing of the solvent residue using rotatory evaporator. The precipitated solid was filtered off, dried and recrystallized from ethanol to give pure **7a–e, 10b,c and 13b,c** in good yields.

#### N'-(7-Chloroquinolin-4-yl)-2-oxo-2H-chromene-3-carbohydrazide (7a)

Red solid, yield (0.37 g, 80%) m.p: 294–296 °C**, FT-IR** (KBr) cm^−1^** ʋ:** 3265 (NH), 1700 (C–C=O), 1655 (C–C=O), 1603 (C=C_Ar_), 1532 (C=N), 1202 (C–C). ^**1**^**H-NMR** δ ppm: 7.44–8.25 (m, 8H, CH_Ar_), 8.42 (s, 1H, CH_Ar_), 8.89 (s, 1H, CH=N_Ar_), 10.82 (br.s, 1H, NH), 14.67 (br.s, 1H, NH). ^**13**^**C-NMR** δ ppm: 116.25, 116.73, 118.36, 119.45, 122.11, 123.71, 125.04, 125.25, 125.69, 125.90, 128.18, 128.79, 130.22, 147.27, 151.97, 153.84. **EI-MS**, m/z (C_19_H_12_ClN_3_O_3_) calcd, 365.06; found, 365.48 [M^+^]=13.36%, 367.56 [M + 2]^+^.

#### N-(2-((7-Chloroquinolin-4-yl) amino) ethyl)-2-oxo-2H-chromene-3-carboxamide (7b)

Yellowish solid, yield (0.46 g, 92%) m.p: 274–276 °C, **FT-IR** (KBr) cm^−1^** ʋ:** 3409 (NH), 2954(CH), 1685 (C–C=O), 1648 (O–C=O), 1610 (C=C_Ar_), 1538 (C=N), 1240 (C–C). ^**1**^**H-NMR** δ ppm: 3.64 (m, 2H, CH_2_), 3.89 (m, 2H, CH_2_), 7.47–8.41 (m, 8H, CH_Ar_), 8.54 (s, 1H, CH_Ar_), 8.91 (s, 1H, CH=N_Ar_), 13.32 (s, 1H, NH). ^**13**^**C-NMR** δ ppm: 29.00, 37.81, 116.13, 117.43, 118.75, 123.96, 124.19, 125.14, 127.49, 130.31, 133.42, 134.16, 147.76, 150.14, 151.89, 153.92, 160.19, 161.70. **EI-MS,** m/z (C_21_H_16_ClN_3_O_3_) calcd, 393.09; found, 393.32[M^+^]=11.18%, 395.43 [M + 2]^+^.

#### N-(3-((7-Chloroquinolin-4-yl) amino) propyl)-2-oxo-2H-chromene-3-carboxamide (7c)

White solid, yield (0.42 g, 81%) m.p: 191–1946 °C**, FT-IR** (KBr) cm^**−**1^** ʋ:** 3331 (NH), 2925(CH), 1710 (C–C=O), 1640 (O–C=O), 1609 (C=C_Ar_), 1532 (C=N), 1213(C–C). ^**1**^**H-NMR** δ ppm: 1.93 (m, 2H, CH_2_), 3.46 (br.m, 2H, CH_2_), 3.64 (br.m, 2H, CH_2_), 7.41–7.97 (m, 8H, CH_Ar_), 8.27 (m, 1H, CH_Ar_), 8.81 (m, 1H, CH=N_Ar_). ^**13**^**C-NMR** δ ppm: 27.66, 37.33, 53.00, 116.26, 117.48, 118.52, 119.19, 124.18, 125.31, 127.04, 130.34, 133.82, 134.24, 147.38, 148.53, 151.59, 153.94, 160.42, 161.53. **EI-MS,** m/z (C_22_H_18_ClN_3_O_3_) calcd, 407.10; found, 407.66[M^+^] = 9.97%, 409.78 [M + 2]^+^.

#### N-(4-((7-Chloroquinolin-4-yl) amino) phenyl)-2-oxo-2H-chromene-3-carboxamide (7d)

Brownish yellow solid, yield (0.47 g, 83%) m.p: 258–2606 °C, **FT-IR** (KBr) cm^−1^** ʋ:** 3390 (NH), 1688 (C–C=O), 1664 (O–C=O), 1609 (C=C_Ar_), 1539 (C=N), 1203 (C–C). ^**1**^**H-NMR** δ ppm: 7.39–8.49 (m, 12H, CH_Ar_), 8.53 (m, 1H, CH_Ar_), 8.93 (s, 1H, CH=N_Ar_), 9.62 (br.s, 1H, NH), 10.73 (s, 1H, NH). ^**13**^**C-NMR** δ ppm: 116.44, 118.00, 118.63, 120.14, 124.17, 124.76, 125.03, 125.85, 126.58, 130.46, 134.87, 135.86, 136.01, 144.70, 147.50, 147.91, 149.40, 152.72, 154.04, 160.62. **EI-MS,** m/z (C_25_H_16_ClN_3_O_3_) calcd, 441.09; found 441.72 [M^+^] = 20.22%, 443.81 [M + 2]^+^.

#### N-(4-(4-((7-Chloroquinolin-4-yl) amino) benzyl) phenyl)-2-oxo-2H-chromene-3-carboxamide (7e)

Yellow solid, yield (0.55 g, 82%) m.p: 251–2566 °C, **FT-IR** (KBr) cm^−1^** ʋ:** 3202 (NH), 1702 (C–C=O), 1660 (O–C=O), 1597 (C=C_Ar_), 1545 (C=N), 1205 (C–C). ^**1**^**H-NMR** δ ppm: 4.02 (m, 2H, CH_2_), 7.35–8.18 (m, 8H, CH_Ar_), 8.25 (m, 4H, CH_Ar_), 8.50 (m, 4H, CH_Ar_),8.81 (m, 1H, CH_Ar_), 8.90 (s, 1H, CH=N_Ar)_, 10.67 (s,1H, NH), 10.90 (br. s, 1H, NH). ^**13**^**C-NMR** δ ppm: 52.09, 116.20, 120.29, 122.19, 124.41, 125.36, 125.88, 126.00, 127.28, 128.21, 128.88, 129.40, 130.29, 135.53, 140.26, 141.46, 148.90, 152.05. **EI-MS,** m/z (C_32_H_22_ClN_3_O_3_) calcd, 531.13; found 531.94 [M^+^] = 11.32%, 533.68 [M + 2]^+^.

#### N-(2-(Acridin-9-ylamino)ethyl)-2-oxo-2H-chromene-3-carboxamide (10b)

Brownish yellow solid, yield (0.4 g, 77%) m.p: ˃300, **FT-IR (KBr) cm**^−1^** ʋ:** 3363(NH), 3390 (NH), 2918(CH), 1698 (C–C=O), 1659 (O–C=O),1611 (C=C_Ar_), 1519 (C=N), 1238 (C–C). ^**1**^**H-NMR** δ ppm: 3.71 (m, 2H, CH_2_), 4.03 (m, 2H, CH_2_), 7.19–8.28 (m, 12H, CH_Ar_), 8.85 (s, 1H, CH_Ar_), 9.19 (s, 1H, NH) 11.75 (s, 1H, NH). ^**13**^**C-NMR** δ ppm: 50.72, 56.01, 116.08, 117.32, 118.44, 118.66, 120.47, 120.97, 122.54, 125.09, 125.99, 130.27, 133.42, 134.07, 140.88, 147.58, 153.87, 160.33, 161.37. **EI-MS**, m/z (C_25_H_19_N_3_O_3_) calcd, 406.14; found 406.01[M^+^] = 10.7%.

#### N-(3-(Acridin-9-ylamino) propyl)-2-oxo-2H-chromene-3-carboxamide (10c)

Brownish yellow, yield (0.44 g, 82%) m.p: 261–263 °C, **FT-IR** (KBr) cm^−1^** ʋ:** 3235 (NH), 2922 (CH),1679 (C–C=O), 1596 (C=C_Ar_), 1531 (C=N), 1260 (C–C). ^**1**^**H-NMR** δ ppm: 1.21 (br.m, 2H, CH_2_), 4.02 (m, 2H, CH_2_), 7.24–7.94 (m, 10H, CH_Ar_), 8.23 (br.m, 2H, CH_Ar_), 8.83(m, 1H, CH_Ar_), 11.78 (s, 1H, NH). ^**13**^**C-NMR** δ ppm: 29.07, 37.01, 116.08, 117.34, 118.43, 118.79, 120.48, 120.99, 122.11, 125.07, 126.00, 130.18, 133.44, 134.07, 140.90, 147.22, 153.82, 161.25, 176.77. **EI-MS,** m/z (C_26_H_21_N_3_O_3_) calcd, 423.16; found 423.10 [M^+^] = 15.84%.

#### N-(2-((5-Methyl-5H-indolo[2,3, b] quinolin-11-yl) amino) ethyl)-2-oxo-2H-chromene -3-carboxamide (13b)

Brown solid, yield (0.5 g, 85%) m.p: 158–160, **FT-IR (KBr) cm**^−1^** ʋ:** 3202(NH), 3327 (NH),1705 (C–C=O), 1659 (O–C=O), 1611 (C=C_Ar_), 1519 (C=N), 1238 (C–C). ^**1**^**H-NMR** δ ppm: 3.65 (br.m, 2H, CH_2_), 4.05 (s, 3H, N-CH_3_), 4.16 (br.m, 2H, CH_2_), 7.06–7.92 (m, 12H, CH_Ar_), 8.51 (m, 1H, CH_Ar_), 12.65 (br. s, 1H, NH). ^**13**^**C-NMR** δ ppm: 32.15, 47.71, 115.72, 116.03, 116.39, 118.08, 118.28, 120.56, 122.21, 123.88, 124.79, 125.09, 130.22, 130.48, 134.10, 137.16, 147.20, 148.65, 152.13, 153.73, 156.29, 159.82, 161.54. **EI-MS,** m/z (C_28_H_22_N_4_O_3_) calcd, 462.17; found 462.53 [M]^+^  = 39.6%.

#### N-(3-((5-methyl-5H-indolo[2,3-b] quinolin-11-yl) amino) propyl)-2-oxo-2H-chromene-3-carboxamide (13c)

Brown solid, yield (0.48 g, 79%) m.p: 158–160 °C, **FT-IR** (KBr) cm^−1^** ʋ:** 3202(NH), 3327 (NH), 2967 (CH), 1705 (C–C=O), 1659 (O–C=O), 1611 (C=C_Ar_), 1519 (C=N), 1238 (C–C). ^**1**^**H-NMR** δ ppm: 1.97(m, 2H, CH_2_), 3.92 (br.m, 2H, CH_2_), 4.16(m, 3H, CH_3_), 7.06–7.92 (m, 12H, CH_Ar_), 8.65 (m, 1H, CH_Ar_). ^**13**^**C-NMR** δ ppm: 32.46, 36.71, 45.34, 115.72, 115,98, 116.09, 118.33, 118.43, 118.58, 120.83, 122.13, 123.72, 123.92, 124.59, 125.10, 130.24, 130.71, 134.03, 137.27, 147.15, 148.46, 153.80, 160.06, 161.20. **EI-MS,** m/z (C_29_H_24_N_4_O_3_) calcd, 476.18; found 476.40 [M^+^] = 24%.

### Bio-evaluation assays

#### Safety assays and anti-proliferative activities of Coumarin hybrids

The safety assays of coumarin hybrids on noncancerous cell lines and their anticancer effects on cancerous cells were gauged utilizing MTT-assay (*Promega*) in accordance with the instruction protocol. Starting from 60 to 200 µM, serial dilutions of coumarin hybrids were prepared in sterile DMSO to be treated cell lines at final concentrations 30 to 100 µM. The treated cells were incubated for 2 days, and the cellular cytotoxicity was detected by quantifying the solubilized formazan in DMSO at 570 nm. The inhibition concentration of 50 (IC_50_) was calculated from the cytotoxicity% curve using *GraphPad prism 9*.

#### Selectivity index of Coumarin hybrids

Cancer cell selectivity index of Coumarin hybrids samples were measured as explained by ^45^, with a minor modification; (SI = IC_50_nc/IC_50_cc), where IC_50_nc refers to the value of IC_50_ of the coumarin hybrids compounds on normal cells, while IC_50_cc refers to the IC_50_ of the coumarin hybrids compounds on cancer cell line**.**

#### Antimicrobial activities of coumarin hybrid

The antimicrobial activity of coumarin hybrids samples were checked against different multiple drug resistant microorganisms (*Escherichia coli*, *pseudomonas aeruginosa, Staphylococcus aureus* and *Streptococcus mutans* and *Candida albicans*) using various concentrations (30 to 100 µM). An aliquot of 100.0 µl of each sample concentration was added to an equal volume of each microbial growth dilution (about 10^6^ CFU/ml*)* and inoculated into 96 well plate. Furthermore, 100.0 µl of LB media was added to 100.0 µl of microbial growth to set as the negative control group. After that, the inoculated plates were incubated overnight at 37 °C then, the microbial turbidity was measured using automated ELIZA microplate reader (BINDER BIOTECK E LX 800) adjusted at 620 nm. The microbial inhibition percentage after treatments were quantified using the following equation:

1 $${\text{Inhibition percentage }} = \, \left( {{\text{A}} - {\text{A1}}/{\text{A}}0} \right) \, \times {1}00.$$

where, A: the treatment group absorbance, A1: the blank absorbance, and A0: the control group absorbance. The Minimal Inhibitory Concentrations (MIC) were estimated and expressed as the lowest concentration of the tested samples which resulted in microbial growth inhibition.

## Ethical statement

All research studies followed the Helsinki World Medical Association's Declaration: Ethical Medical Research Principles Involving Human Subjects and were approved by the ethics committee at Menoufia University in Egypt, Faculty of Science. Where, no animal experiments were conducted in this research, while all used cell lines were obtained from ATCC with original and recognized serial number of each cell line as aforementioned in section of cell line and microbial strains.

## Results and discussion

### General synthesis of coumarin- N-heterocyclic hybrids

#### Synthesis of coumarin-3-carbonyl chloride

**Scheme 1:**
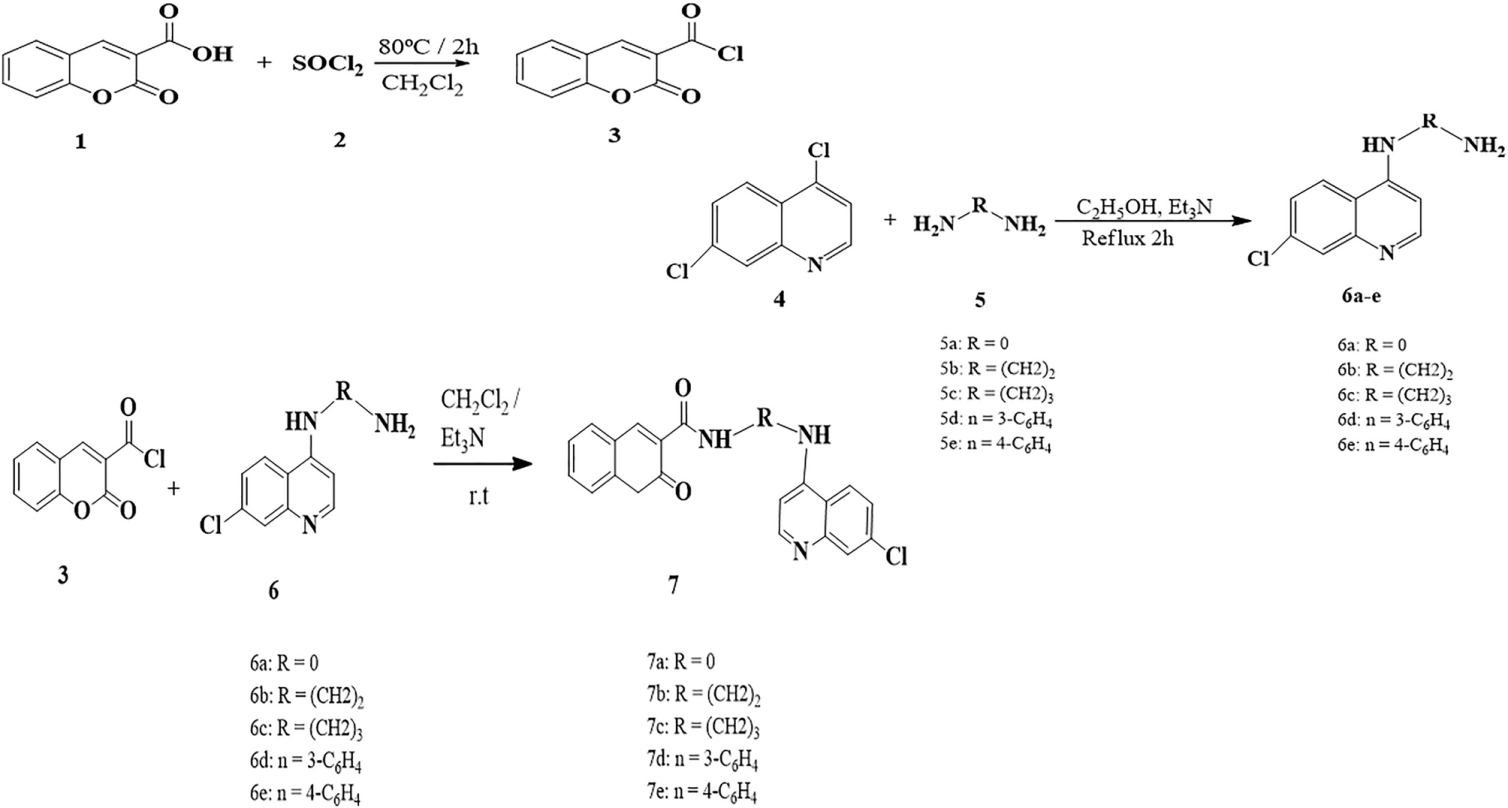
Synthetic pathway of coumarin-3-carbonyl chloride; synthesis of 4-bis amino quinoline **6a–e** and synthesis of coumarin hybrids **7a–e**.

The key intermediate acid chloride **3** was prepared in good yield as pale-yellow crystals according to published method^21,46,47^, by the reaction of coumarin-*3*-carboxylic acid **1** and thionyl chloride (SOCl_2_**)** refluxed for 2 h as depicted in Scheme 1.

#### Synthesis of 4 bis-aminosubstituted heterocycles

The reaction of 4,7-dichloroquinoline **4** with hydrazine, aliphatic and aromatic diamines **5a–e** in presence of triethyl amine as a base catalyst afforded **6a-e** in good yields via nucleophilic aromatic substitution (S_NAr_). The synthesized compounds showed analytical data consistent with previously published results^4,48^.

**Scheme 2:**
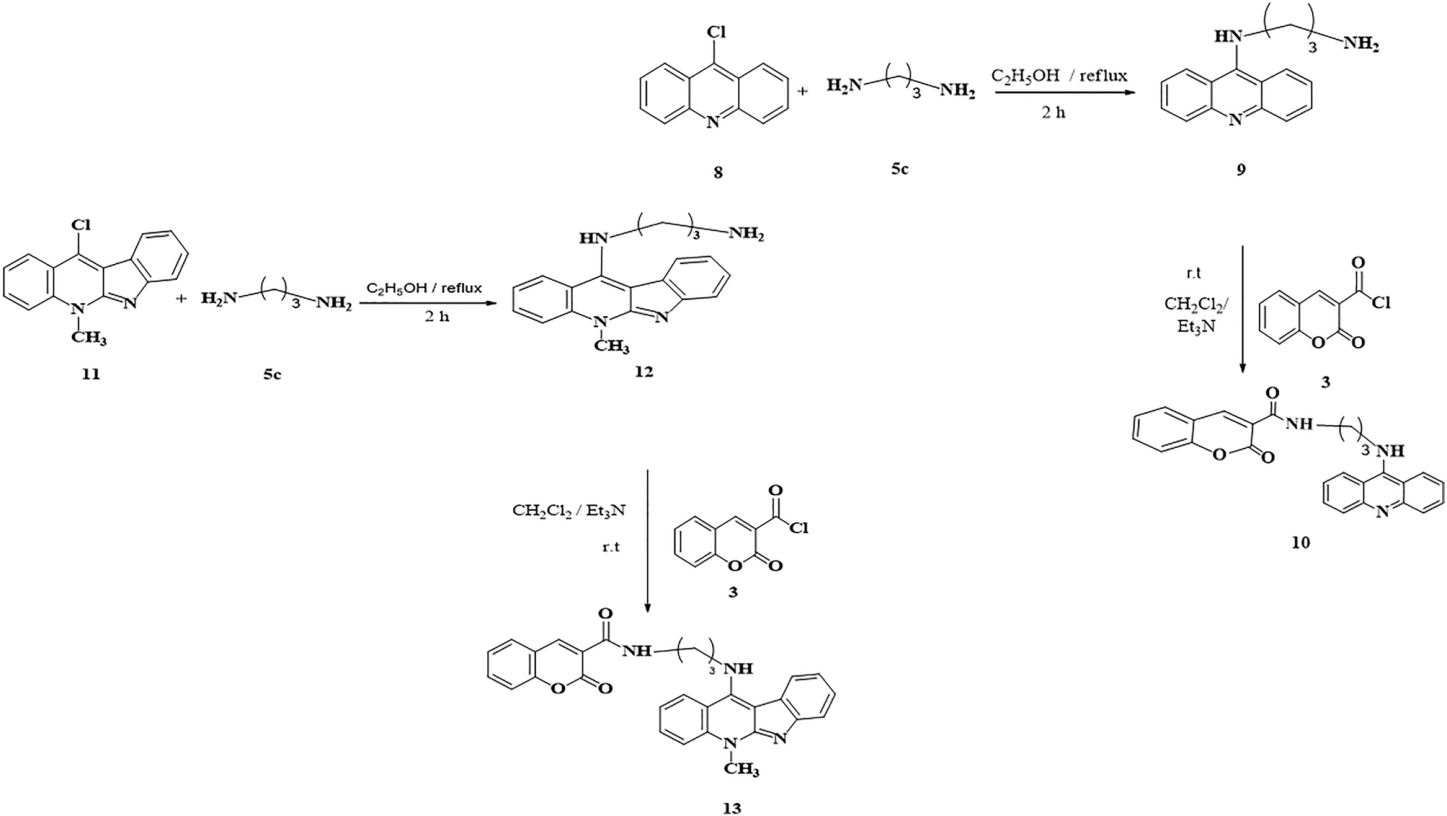
Synthesis of coumarin-acridine hybrid **10***,* and synthesis of coumarin-neocryptolepine hybrid **13.**

The synthetic pathway for formation of coumarin-quinoline hybrids **7a-e** was achieved by the reaction of **3** with diamines **6a-e,** in equimolar ratio in presence of triethyl amine as a base to afford the corresponding hybrids **7a-e,** in good to excellent yields (80–92%) as given in Scheme 1. Additionally, the target hybrids **10b,c** were synthesized by the condensation of 9-chloroacridine **8** with amines **5b, 5c** afforded the **9b** and **9c**, according to the reported method.^49,50^ Further reactions of **9b,c** with **3** in equimolar ratio in presence of excess of triethyl amine in good yield (77,82%) as depicted in Scheme 2.

Furthermore, the condensation of 11-chloroneocryptolpine **11** with **5b** and **5c** yielding **12b,c** in good yield (79%) according to the reported methods^51,52^. While, the reaction of **12b, 12c** with **3** in equimolar ratio in presence of excess of triethyl amine afforded the corresponding coumarin-neocryptolepine hybrids **13b,c** in good yields (79,85%), as shown in scheme 2. The progress of reactions was easily monitored by TLC using benzene and ethanol as eluent mixture (3:1), till starting materials are consumed.

#### Chemical structure verification by FTIR and NMR analysis

*FTIR analysis of coumarin hybrids* In structure elucidation of **7 a-e, 10b,c** and **13b,c**, FT-IR spectra show a strong absorption band for ʋ _(NH)_ at: 3265, 3409, 3331, 3390 and 3390 cm^−1^ for **7a-e**; respectively, 3235,3390 cm^−1^ for **10b,c** , 3202, 3327 and 3372 cm^−1^ for** 13b,c**. Moreover, the absorption band for ʋ (C–C=O) at 1700, 1685, 1710, 1688 and 1702 cm^−1^ for **7a-e** respectively, 1679,1698 cm^−1^ for **10b,c**, and 1705 cm^−1^ for **13b,c** which confirmed the presence of carbonyl group. On the other hand, the absorption band for ʋ (O–C=O) at ʋ: 1655, 1648, 1640, 1664 and 1660 cm^−1^ for **7 a–e**; respectively, 1659 cm^−1^ for **10b**, 1659 and1650 cm^−1^ for **13b,c** which confirmed the presence of lactone. Moreover, the absorption band for ʋ (C=C_Ar_) at 1603, 1610, 1609, 1609, and 1597 cm^−1^ for **7a-e**; respectively,1596, 1611 cm^−1^ for **10b,c** 1611 and 1609 cm^−1^ for **13b,c.** On the other hand, the absorption band for ʋ (C=N) at: 1532, 1538, 1532, 1539 and 1545 cm^−1^ for **7a-e**; respectively, 1531,1568 cm^−1^ for **10b,c**, 1519 and1538 cm^−1^ for **13b,c**. Furthermore, the absorption band for ʋ (C–C) at: 1202, 1240, 1213, 1203, and 1205 for **7a-e**; respectively, 1238 cm^−1^ for **10b,c**, 1238 and 1245 cm^−1^ for **13b,c.**

*NMR analysis of coumarin hybrids* ^1^H-NMR spectrum shows multiple δ 3.46 and 3.89 ppm which correspond to the aliphatic spacers in **7b**. Moreover, the spectra showed multiple with δ: 1.93 ppm, broad multiple with δ: 3.46 and 3.64 ppm, that confirmed the presence of aliphatic spacer in **7c**. On the other hand, spectra showed multiple with δ: Moreover, the spectra showed multiple with δ: 4.02 ppm which confirmed the presence of (Ph-CH_2_-Ph) for **7e**, showed broad multiple with δ: 4.16, 4.05 for which confirmed the presence of (*N*-CH_3_) for **13b,c**. On the other hand, coumarin (CH_Ar_) appear with δ: 8.42, 8.54, 8.26, 8.53, 8.81 ppm for **7a–e**; respectively, 8.83, 8.85 ppm for **10b,c,** 8.65 and 8.51 ppm for **13b,c**. Furthermore, (HC=N_Ar_) appear with δ: 8.89, 8.91, 8.81, 8.93 and 8.90 ppm for **7a–e** respectively**.** Moreover**,** the spectra showed broad singlet with δ: (NH) 10.82, 14.67, 13.32, 9.62, 10.73, 10.67, 10.90 ppm for **7a–e**, and 9.19, 11.75, and 11.78 ppm for **10b,c** and 12.65 ppm for **13b**.

While ^**13**^**C-NMR** spectra showed Peak of (*N*-CH_3_) for **13b,c** at 32,46 and 32.15 ppm. On the other hand, the coumarin (HC=C) appears at: δ = 119.45, 118.75, 119.19, 120.14, 120.29 ppm respectively for **7a–e**, 120,99 120.47 ppm for **10** and 120,83, 120.56 ppm for **13.** Moreover, lactone (O–C=O) appear at δ: 151.97, 160.19, 160.42, 154.04, 152.05 ppm; respectively for **7a–e**, 161.25, 160.33 ppm for **10b,c** and 159.82,160.06 ppm for **13b,c.** Furthermore, the carbonyl group (C–C=O) appears at δ: 153.84, 161.70, 161.53, 160.62, 152.05 ppm for **7a–e**; respectively, 176.77,161.37 ppm for **10b,c** and 161,20, 161.54 ppm for **13b,c**. on the other hand, for the mass spectra for synthesized hybrids showed coincidence of the molecular ion peak with their expected molecular weight.

### Bioevaluation assessment

#### Safety assays and anticancer activities of coumarin hybrids compounds

**Figure 2:**
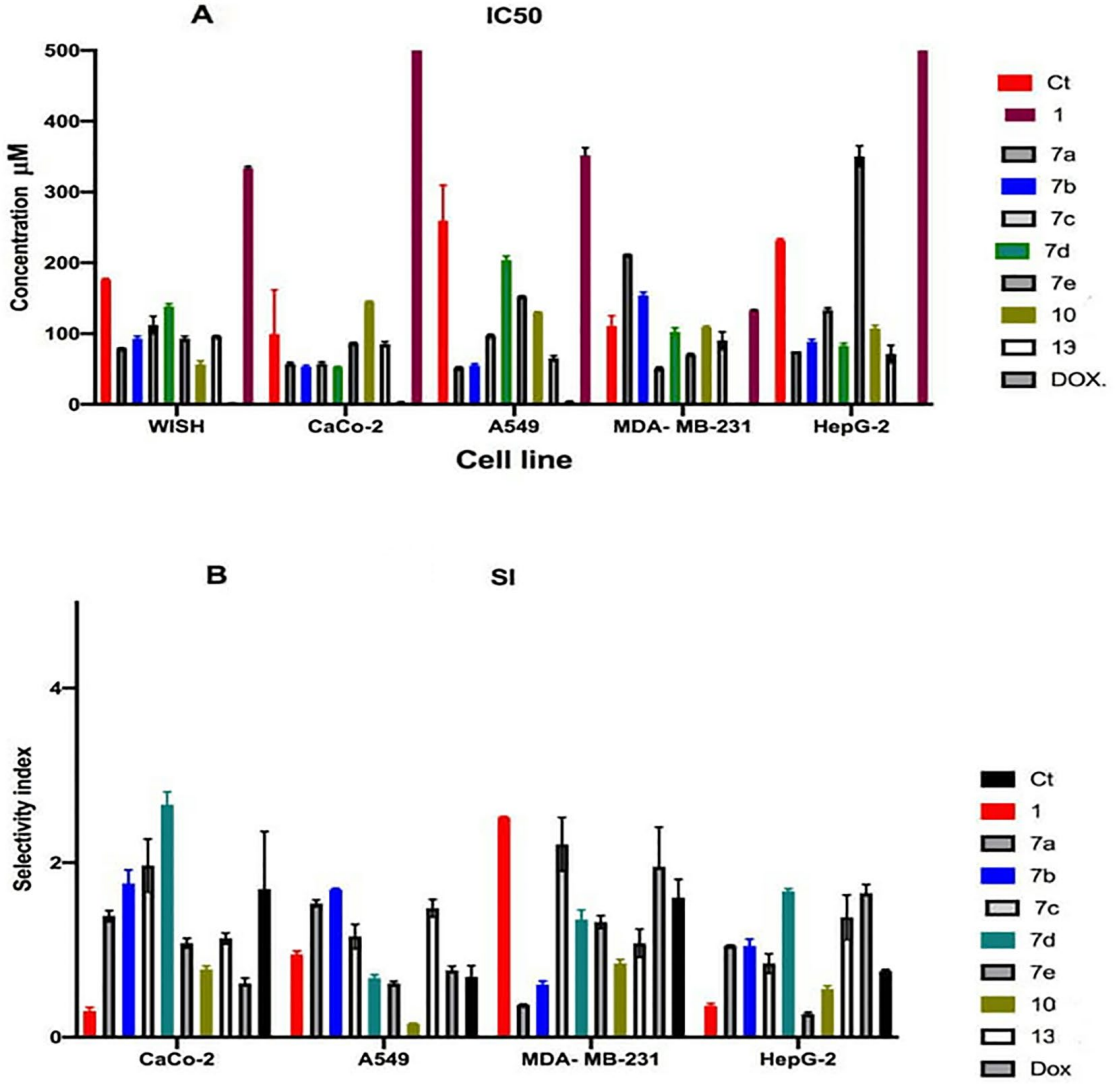
IC_50_ (**A**) and SI (**B**) values of tested synthesized coumarin hybrid compounds.

The safety profiles on *WISH* cells and the anticancer potentialities of coumarin hybrids compounds against *MDA-MB-231, CaCo-2, A549* and *HepG-2* cell lines were measured utilizing MTT-assay (Fig. 2A,B). The concluded data indicated that the compounds **7c, 7d** and **13c** were the safest compounds on *WISH* cells with IC_50_ values of 112.39, 138.39 and 95.88; respectively (Table 1). While, among the control samples, **1** and coumarin (**Ct**) samples were safer than the other coumarin hybrids compounds with IC_50_ values of 333.57 and 176.36 µM; respectively (Fig. 2A and Table 1).

**Table 1:** IC_50_ values of measured coumarin hybrid compounds compared to DOX as reference drug.

Cell line	Ct	**1**	**7a**	**7b**	**7c**	**7d**	**7e**	**10**	**13**	DOX
WISH	176.36	333.575	79.2825	92.92	112.39	138.39	93.065	56.744	95.88	1.7635
CaCo-2	99.46	1111.5	57.1	52.785	57.29	51.95	86.31	144.775	84.755	2.91
A549	259.67	351.475	51.72	54.815	97.395	203.99	151.87	130.097	65	3.777
MDA-MB-231	111.19	132.32	211.315	154.04	50.965	102.625	70.54	108.947	89.94	0.655
HepG-2	231.67	924.325	75.42	88.69	133.11	82.835	350.645	107.197	70.95	1.175

On the other hand, the most active compounds showed different anti-proliferative profiles on different cancerous cell lines. For example, **7c** and **7d** were the most effective compounds against *CaCO-2* cell line with IC_50_ values of 57.29 and 51.59 µM, with anticancer selectivity index 1.9 and 2.6; respectively (Fig. 2A). On *A549* cell lines, the treatments **7a, 7b** and **13c** were the most significant treatments with selectivity index of 1.5, 1.6 and 1.4; respectively (Table 2). Also, **7c** was the most potent treatment against *MDA-MB-231* cell line with SI 2.2 (Fig. 2B). Furthermore, **7d** was the most potent compound against *HepG-2* cell line with IC_50_ 82.83 µM and SI 1.67 (Fig. 2A).

**Table 2:** Selective index values of measured coumarin hybrid compounds.

Cell line	Ct	1	7a	7b	7c	7d	7e	10	13	DOX
CaCo-2	1.7005019	0.30274004	1.38978276	1.76403734	1.96871657	2.66600438	1.07865591	0.77853734	1.13263098	0.61475703
A549	0.69149983	0.94968866	1.53332834	1.69539443	1.15483508	0.67892756	0.61291219	0.153999	1.47786267	0.76533619
MDA- MB-231	1.59929755	2.52101246	0.37518942	0.6037819	2.21008859	1.35138147	1.32002314	0.84618234	1.07704405	1.95350877
HepG-2	0.76134548	0.36167827	1.05121285	1.04899673	0.84562991	1.67133135	0.26583552	0.55325474	1.37354783	1.65253623

#### Antimicrobial activities of coumarin hybrids

The antimicrobial potency of coumarin hybrids was tested against different multiple drug resistant strains using microplate assay method. The results indicated that all investigated hybrids showed positive effects against all investigated microbes.

**Figure 3:**
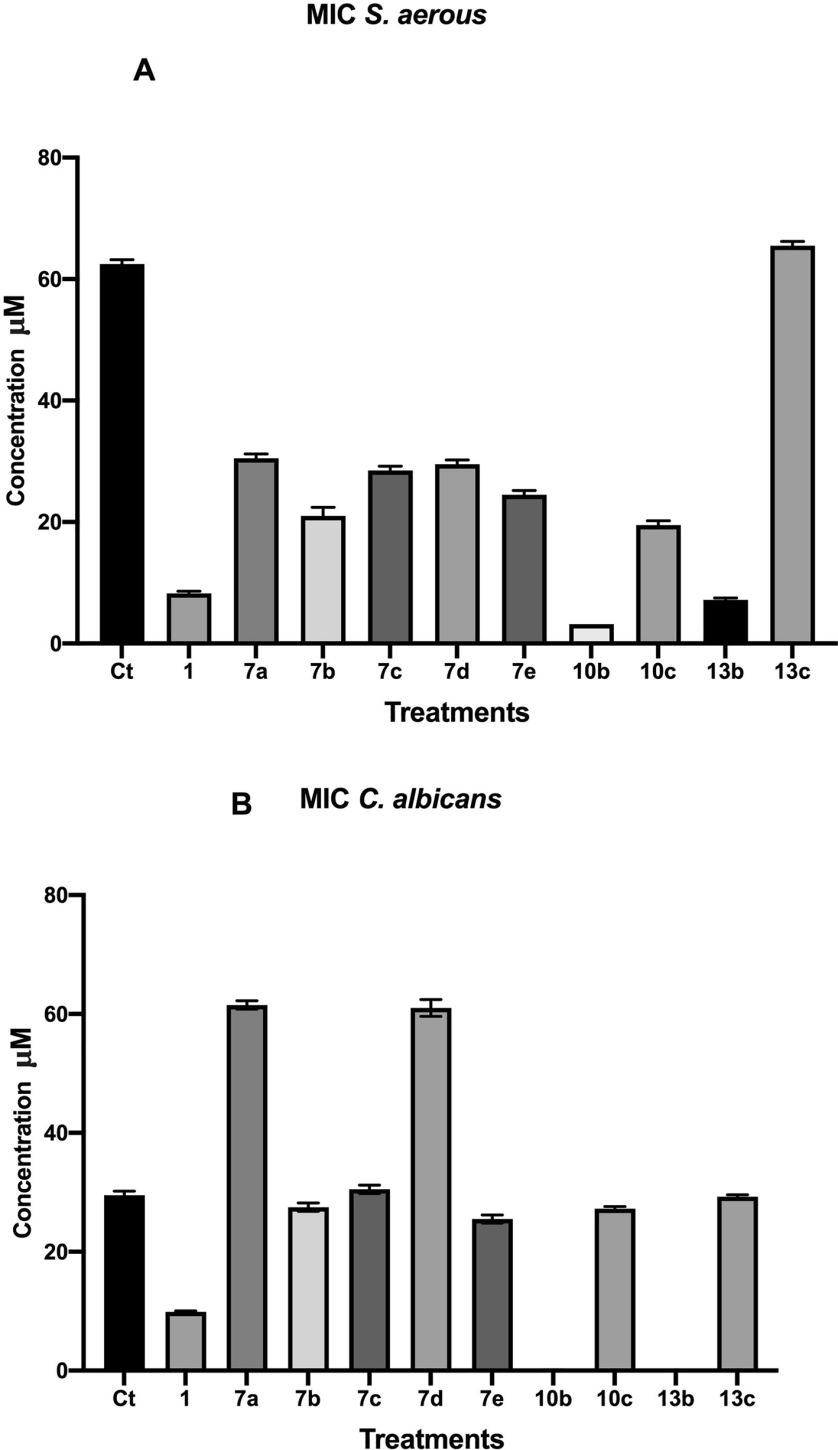
MIC values of the synthesized coumarin hybrid compounds against *Staphylococcus aureus* and *Candida albicans*.

**Figure 4:**
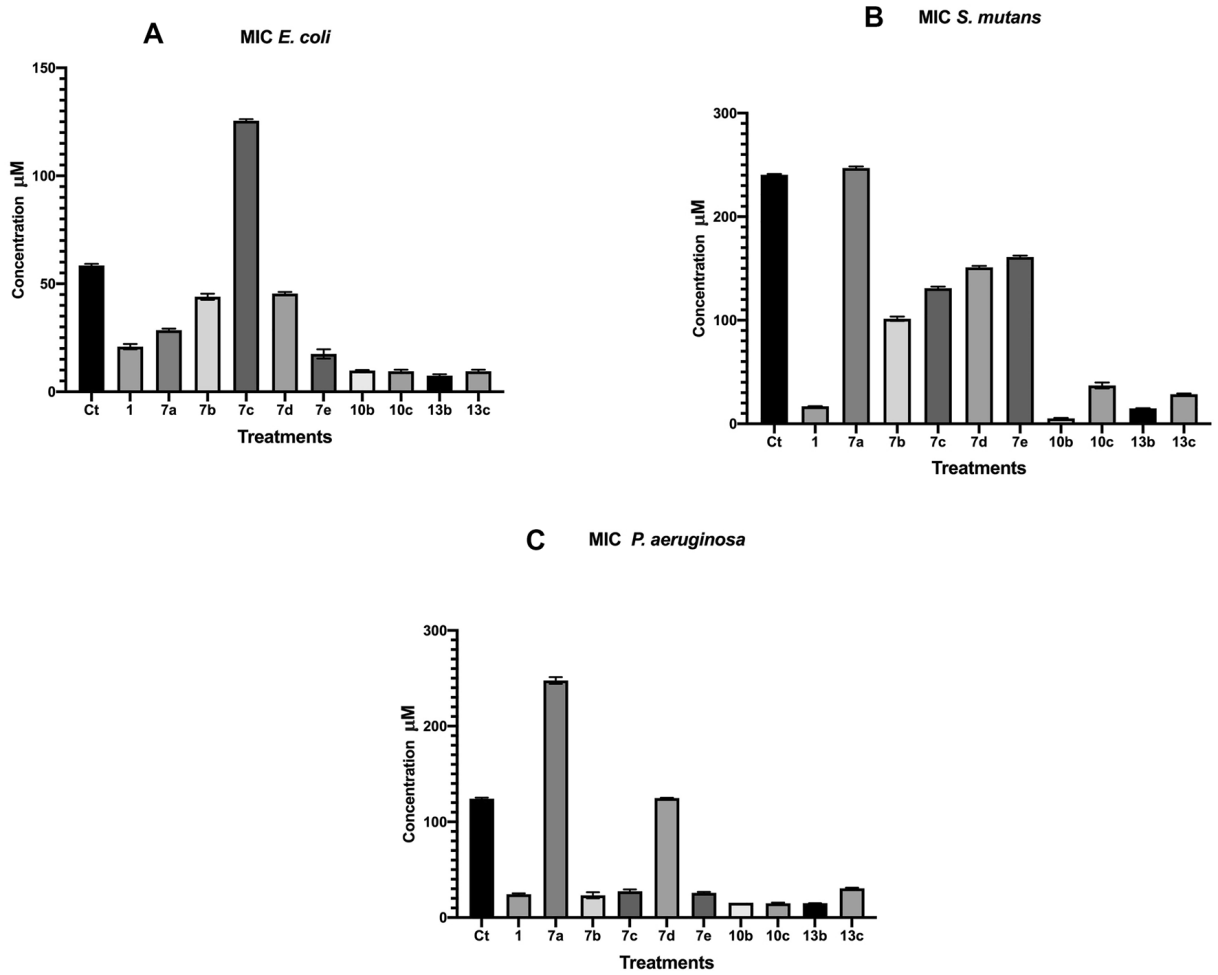
MIC values of the synthesized coumarin hybrid compounds against *Pseudomonas aeruginosa Streptococcus mutans* and* E. coli.*

Furthermore, samples **10b** and **10c** considered as the most efficient treatment against *p. aeruginosa* with MIC value of 14.8 and 15.4 µM, respectively (Table 3). Also, against both *C. albicans, S. aureus* samples **10c** and **13c** were the most potent treatments with MIC values of 0.0011 and 0.0013 µM, and 3.2 and 7.2 µM, respectively (Fig. 3). Concerning the antimicrobial activity against *E. Coli,* **10b**, **13b** and **13c** were the most potent compounds with MIC values of 9.5, 9.8 and 9.4, respectively (Fig. 4). Finally, **10c**, **13c** and **13b** were the most effective against *Strep. mutans* with a MIC value of 5.2, 14.8 and 28.5μM, respectively (Table 3).

**Table 3:** MIC values of the synthesized coumarin hybrids against *Staphylococcus aureus, Candida albicans, E. coli, Streptococcus mutans* and *Pseudomonas aeruginosa*.

	**Ct**		**1**		**7a**		**7b**		**7c**		**7d**		**7e**		**10b**		**10c**		**13b**		**13c**	
*Staph. aureus*	62	63	8	8.5	30	31	20	22	29	28	29	30	25	24	3.2	3.2	20	19	7	7.4	65	66
C. *albicans*	30	29	10	9.8	62	61	28	27	30	31	62	60	25	26	0.12	0.13	27	27.5	0.0011	0.0014	29	29.5
*E. coli*	59	58	20	21.76	29	28	45	43	126	125	46	45	19	16	10	9.7	10	9	7	7.88	10	9
S. *mutans*	240	241	17	16.65	248	246	100	103	150	152	130	132	160	162	5	5.5	39	35	15	14.67	29	28

#### Molecular docking studies

The anticancer effect of coumarins is assumed to be attributed to a variety of mechanisms, such as regulation of the estrogen receptor, activation of cell death, blockage of the cell cycle, and inhibition of DNA-associated enzymes including telomerase and topoisomerase (TOP).^36,53,54^ A significant portion of fundamental cellular biology involves DNA topoisomerases, which are also molecular targets for several medications, including antibiotics, antibacterials, and anticancer medications. They work by preventing the topoisomerase molecule from relegating DNA strands upon cleavage, so changing it into an agent that damages DNA^55,56^. In the field of medicinal chemistry and drug development, hybrid molecules which are created by combining two or more pharmacophores are a relatively new idea that has gained a lot of attention lately. Therefore, hybridization of coumarin with other anticancer pharmacophores may provide novel bioactive candidates with potential activity as well as low cytotoxicity. The design approach involves the coupling of coumarins with other bioactive molecules as neocryptolepine a quinoline based compound- aiming to afford anticancer compounds that might target topoisomerase.^57–60^.

**Figure 5:**
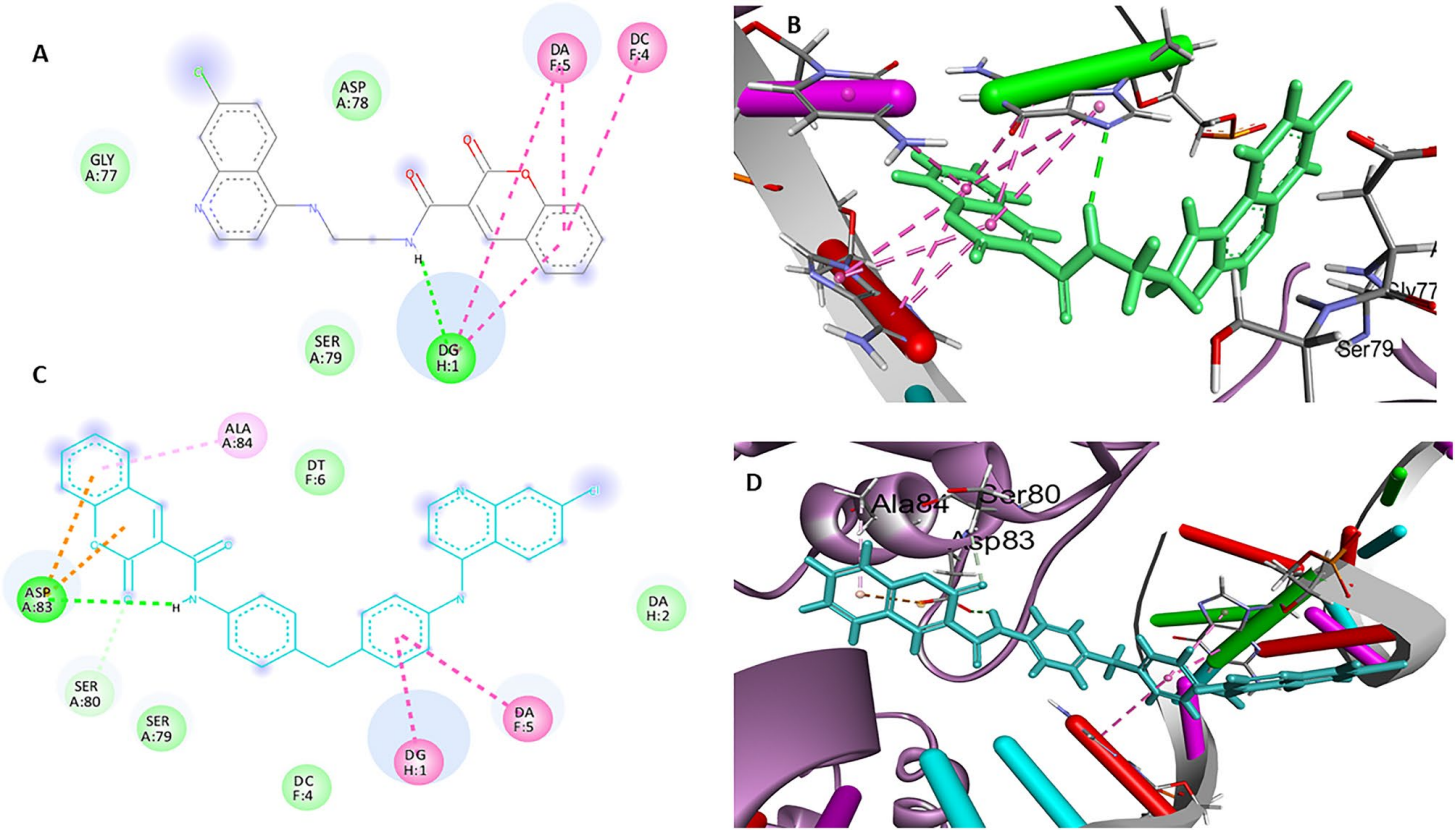
(**A–D**) Docking results of the synthesized compounds, best scoring compounds’ interactions **7b** (in green sticks) and **7e** (in cyan sticks) inside the active site of topoisomerase 2 with H-bonds in green, Pi-Pi stacking in pink, pi-H bonds in light pink and pi anion bonds in orange color.

*In-silico* screening of the quinoline based compounds revealed their promising affinity to Topoisomerase II which is consistent with studies done on similar compounds as acridine and neocryptolipines^61^. The proposed binding mode of the investigated compounds showed marked affinity values ranging from a maximum value of -6.5392 kcal/mol recorded by **7e** and a minimum value of -5.5638 kcal/mol recorded by **7c**. Docking results disclosed that the planar di or tri ring aromatic system showed *pi*-hydrophobic or π-π interactions mostly with the amino acids residues *Asp78, Asp83, Ser79* while, DG1, DC4, and DG5 were the residues for interaction at the DNA-minor groove, (Fig. 5). These interaction are consistent with the reported interactions by the co-crystallized ligand “**7-[(3R)-3-aminopyrrolidin-1-yl]-8-chloro-1-cyclopropyl-6-fluoro-4-oxo-1,4-dihydro quinoline-3-carboxylic acid**” in the key residues *Ser79* as well as DG1 in the DNA groove.^44^ The synthetic compounds' docking results include the binding affinity Score and Root Mean Square Deviation (RMSD). The following table lists the ligand interactions with the active site residues, including hydrogen bonding and hydrophobic interactions (Table 4).

**Table 4:** Receptor binding affinity, RMSD values and residues involved in the interaction at the receptor active site.

Comp. No	Binding energy Kcal/mol	RMSD (A)	Amino acids involved and type of interactions
7a	− 6.0373	1.9272	Asp78(H-bond), DA5(π–π)
7b	− 6.4308	1.4711	DG1(H-bond), DG1(*π*–*π*), DC4(π–π), DA5(π–π)
7c	− 5.5638	2.1466	DG1(π–π)
7d	− 5.8557	1.9477	D Asp78(*pi*-H)
7e	− 6.5392	1.6699	DG1(π–π), DA5(π–π), Asp83(H-bond, pi-anion), Ala 84(*pi*-H)
10	− 5.8972	1.5769	DG1(π–π)
13	− 6.3032	1.2402	DG1(π–π), Asp78(*pi*-H), Ser79(*pi*-H)

#### In-silico ADME assessment

The synthesized compounds were put through an in-silico investigation to determine their pharmacokinetic characteristics using Swiss ADME^62^. The investigated compounds demonstrated high GI absorption ability except for compound **7e**. Moreover, the tested hybrids show low Blood Brain Barrier (BBB) permeability (Table 5). Cell membrane penetrability reflected by the log *P* values, range from 2.77 to 3.91, these values are < 5 which reflects marked cell membranes tolerability^63^. Following “rule of five” for Lipinski, all compounds’ molecular weights were less than 500 except for compound **7e** which was slightly higher than 500 recording value of 531.99. In addition, Hydrogen-Bond Donors (HBD) were 2 and Hydrogen-Bond Acceptors (HBA) were 4 for all tested compounds, with rotatable bonds ranging from 4 to 7. The bioavailability score was 0.55 for all compounds except **7e** which recorded the lowest bioavailability score 0.17, this might be attributed to its high molecular weight. All screened compounds had promising ADME data that revealed compliance with the Lipinski’s rule except for compound **7e** that had two *Lipinski’s* violations with high molecular weight and low bioavailability score which affected its GI absorption probabilities (Table 6).

**Table 5:** In-silico screening for ADME properties of compounds 7a–d,10 and 13.

Comp. No.	MW	No. rotatable bonds	No. H-bond acceptors	No. H-bond donors	MR	iLOGP	GI absorption	BBB	Bioavailability score	Lipinski violations
7a	365.77	4	4	2	99.81	2.77	High	Low	0.55	0
7b	393.82	6	4	2	109.42	3.19	High	Low	0.55	0
7c	407.85	7	4	2	114.23	3.01	High	Low	0.55	0
7d	441.87	5	4	2	126.55	3.45	High	Low	0.55	0
7e	531.99	7	4	2	156.01	3.78	Low	Low	0.17	2
10	423.46	7	4	2	126.73	3.18	High	Low	0.55	0
13	476.53	7	4	2	143.49	3.91	High	Low	0.55	0

**Table 6:** In silico screening for ADME properties of compounds 7a–d,10c and 13c.

	MW	No. rotatable bonds	No. H-bond acceptors	No. H-bond donors	MR	iLOGP	GI absorption	BBB	Bioavailability score	*Lipinski* violations
7a	365.77	4	4	2	99.81	2.77	High	Low	0.55	0
7b	393.82	6	4	2	109.42	3.19	High	Low	0.55	0
7c	407.85	7	4	2	114.23	3.01	High	Low	0.55	0
7d	441.87	5	4	2	126.55	3.45	High	Low	0.55	0
7e	531.99	7	4	2	156.01	3.78	Low	Low	0.17	2
10c	423.46	7	4	2	126.73	3.18	High	Low	0.55	0
13c	476.53	7	4	2	143.49	3.91	High	Low	0.55	0

#### Structural activity relationship

The main objective of the installation of new hybrids containing coumarin and heteroarenes bearing two, three and four fused cyclic rings is to optimize and reach the best activity depending on the synergistic effect of the utilized precursors. Notably, the acridine and neocryptolepine pharmaceutical cores have quinoline motif fused with benzyl as showed in acridine or indole in case of neocryptolepine. Relying on the and from the reported IC_50_ values in Table 2, structure activity relationship of the synthesized hybrids was studied. Table 1 emphasized the presence of anticancer activity of these hybrids comparing to their starting core used as positive control and revealed the synergistic effect of these hybrids. In light of the afforded results of the synthesized hybrids against *MDA-MBA-231* cancer cell line, it is noteworthy that hybrid **7c** containing coumarin and quinoline cores as two fused rings with presence of three carbon spacers of quinolone core showed the best activity with IC_50_:50.96 µM and SI:2.2 folds among of their relatives presenting in hybrids 7**a–e**. Furthermore, it is noteworthy that hybrid **7c** containing two fused rings with propyl spacer at C-4 of quinoline core had higher activity than tetra- and tri cyclic ring belonging to neocryptolepine and acridine cores with the same spacer attached on C-11 and C-9 resembled to **13c** and **10c** respectively. On the other hand, hybrid **13c** showed antiproliferative activity against *HepG-2* cell line compared to their relative hybrids **10c** and **7c.** In addition to, IC_50_ of A549 cell line illustrated that **13c** have the highest potency than **7c** and **10c**. Furthermore, hybrid **7d** showed the best activity against *CaCO-2* cell line which have phenylene diamine as spacer with two fused rings. That illustrated that length of spacer influences the antiproliferative activity. In addition **10b** and **13b**, which contained two carbons spacer with tri and tetra fused rings, showed higher antibacterial and antifungal activity against *S.aureus, s.mutans, E.coli* and *C.albicans*.

## Conclusions

In the current study, hybrids of coumarin‐quinolines **7a–e**, coumarin‐acridines **10b,c** and coumarin- neocryptolepines **13b,c** were synthesized and evaluated for their antiproliferative and antimicrobial activities. Hybrids **7c** and **7d** were proved to be the most potent as an antiproliferative agents, showing inhibitory activity against *MDA-MB-231* and *CaCo-*2 with IC_50_ of 50.96 and 51.95 µM respectively. Furthermore, Hybrids **10b** and **13b** showed higher antibacterial activity against *S.aureus, S.mutans and E. coli* with MIC from 3.2 to 15 µM compared to their corresponding derivatives **7a–e** and **10c** and **13c**. as well as potent inhibitory activity against Candida albicans with MIC 0.0011 to 0.12 µM. Molecular docking study disclosed that the planar di or tri ring aromatic system showed hydrophobic interactions at the binding site of action mostly with the amino acids residues *Asp78, Asp83, Ser79* and DG1, DC4, and DG5 residues at the DNA-minor groove. These interactions are consistent with the reported interactions by the co-crystallized ligand of topoisomerase protein in the key residue *Ser79* and in DG1 of the DNA groove*.* All screened compounds showed promising ADME data following Lipinski’s rule except for compound **7e** that had two Lipinski’s violations with high molecular weight and low bioavailability score which affected its GI absorption probabilities. Overall, the results of this study support the possibility of using these coumarin hybrids as promising anticancer and antimicrobial agents for further in vivo animal model study.

### Supplementary Information

Supplementary Information.

## Data Availability

All raw data of measurements is available and could be shared when requested, both corresponding authors (I.E.T. and E.A.K.) are fully responsible for providing all data requested.

## References

[1] Amjad E, Sokouti B, Asnaashari S (2022). An investigation of 6-Shogaol effects on MCF7 cell lines through a systems biology approach. Egypt. J. Med. Hum. Genet..

[2] Gilad Y, Gellerman G, Lonard DM, O’Malley BW (2021). Drug combination in cancer treatment—From cocktails to conjugated combinations. Cancers.

[3] Bhattarai N, Kumbhar AA, Pokharel YR, Yadav PN (2021). Anticancer potential of coumarin and its derivatives. Mini. Rev. Med. Chem..

[4] Elmongy EI, Ahmed AA, El Sayed IET, Fathy G, Awad HM, Salman AU, Hamed MA (2022). Synthesis, biocidal and antibiofilm activities of new isatin-quinoline conjugates against multidrug-resistant bacterial pathogens along with their in silico screening. Antibiotics.

[5] Dimova I, Popivanov G, Djonov VG (2014). Angiogenesis in cancer-general pathways and their therapeutic implications. Jbuon.

[6] Bhat, S., Muthunatarajan, S., Mulki, S. S., Archana Bhat, K., & Kotian, K. H. Bacterial infection among cancer patients: Analysis of isolates and antibiotic sensitivity pattern. *Int. J. Microbiol*. (2021). 10.1155/2021/8883700 PMC7825358 33510793

[7] Kraljević TG, Harej A, Sedić M, Pavelić SK, Stepanić V, Drenjančević D, Talapko J, Raić-Malić S (2016). Synthesis, in vitro anticancer and antibacterial activities and in silico studies of new 4-substituted 1, 2, 3 triazole–coumarin hybrids. Eur. J. Med. Chem..

[8] Felício M, Silva O, Gonçalves S, Santos N, Franco O (2017). Peptides with dual antimicrobial and anticancer activities. Front Chem.

[9] Diaconu D, Antoci V, Mangalagiu V, Amariucai-Mantu D, Mangalagiu II (2022). Quinoline imidazole/benzimidazole derivatives as dual-/multi-targeting hybrids inhibitors with anticancer and antimicrobial activity. Sci. Rep..

[10] Mantu D, Antoci V, Moldoveanu C, Zbancioc G, Mangalagiu II (2016). Hybrid imidazole (benzimidazole)/pyridine (quinoline) derivatives and evaluation of their anticancer and antimycobacterial activity. J. Enzyme Inhibit. Med. Chem..

[11] Bansal Y, Silakari O (2014). Multifunctional compounds: Smart molecules for multifactorial diseases. Eur. J. Med. Chem..

[12] Sangani CB, Makawana JA, Zhang X, Teraiya SB, Lin L, Zhu H-L (2014). Design, synthesis and molecular modeling of pyrazole–quinoline–pyridine hybrids as a new class of antimicrobial and anticancer agents. Eur. J. Med. Chem..

[13] Keri RS, Budagumpi S, Balappa Somappa S (2022). Synthetic and natural coumarins as potent anticonvulsant agents: A review with structure–activity relationship. J. Clin. Pharm. Therapeut..

[14] Wang X, Zhou H, Wang X, Lei K, Wang S (2021). Design, synthesis, and in vivo and in silico evaluation of coumarin derivatives with potential antidepressant effects. Molecules.

[15] Khan MS, Agrawal R, Ubaidullah M, Hassan MI, Tarannum N (2019). Design, synthesis and validation of anti-microbial coumarin derivatives: An efficient green approach. Heliyon.

[16] Sahoo J, Mekap SK, Kumar PS (2015). Synthesis, spectral characterization of some new 3-heteroaryl azo 4-hydroxy coumarin derivatives and their antimicrobial evaluation. J. Taibah Univ. Sci..

[17] Jouda J-B, Mbazoa CD, Sarkar P, Bag PK, Wandji J (2016). Anticancer and antibacterial secondary metabolites from the endophytic fungus Penicillium sp CAM64 against multi-drug resistant Gram-negative bacteria. Afr. Health Sci..

[18] Tiwari SV, Seijas JA, Vazquez-Tato MP, Sarkate AP, Karnik KS, Nikalje APG (2018). Ionic liquid-promoted synthesis of novel chromone-pyrimidine coupled derivatives, antimicrobial analysis, enzyme assay, docking study and toxicity study. Molecules.

[19] Al-Majedy Y, Al-Amiery A, Kadhum AA, BakarMohamad A (2017). Antioxidant activity of coumarins. Syst. Rev. Pharm..

[20] Wei Y, Li S-Q, Hao S-H (2018). New angular oxazole-fused coumarin derivatives: Synthesis and biological activities. Nat. Prod. Res..

[21] Onar HÇ, Hasniye Y, Oktay S (2019). Comparison of antioxidant activities of mono-, di-and tri-substituted coumarins. J. Turk. Chem. Soc. Sect. A Chem..

[22] Bansal Y, Sethi P, Bansal G (2013). Coumarin: A potential nucleus for anti-inflammatory molecules. Med. Chem. Res..

[23] Park S-H, Sim Y-B, Kang Y-J, Kim S-S, Kim C-H, Kim S-J, Lim S-M, Suh H-W (2013). Antinociceptive profiles and mechanisms of orally administered coumarin in mice. Biolog. Pharm. Bull..

[24] Wu Y, Xu J, Liu Y, Zeng Y, Wu G (2020). A review on anti-tumor mechanisms of coumarins. Front. Oncol..

[25] Thomas V, Giles D, Basavarajaswamy PMG, Kumar Das A, Patel A (2017). Coumarin derivatives as anti inflammatory and anticancer agents. Curr. Med. Chem. Anticancer Agents.

[26] Rawat A, Reddy AVB (2022). Recent advances on anticancer activity of coumarin derivatives. Eur. J. Med. Chem. Rep..

[27] Leal LKAM, Silva AH, Viana GSdB (2017). Justicia pectoralis, a coumarin medicinal plant have potential for the development of antiasthmatic drugs?. Rev. Bras. Farmacognosia.

[28] Hassan MZ, Osman H, Ali MA, Ahsan MJ (2016). Therapeutic potential of coumarins as antiviral agents. Eur. J. Med. Chem..

[29] Al-Majedy, Y. K., Kadhum, A. A. H., Al-Amiery, A. A., & Mohamad, A. B. Coumarins: The antimicrobial agents. *Syst. Rev. Pharm. 8*(1) (2017).

[30] Weigt S, Huebler N, Strecker R, Braunbeck T, Broschard TH (2012). Developmental effects of coumarin and the anticoagulant coumarin derivative warfarin on zebrafish (Danio rerio) embryos. Reprod. Toxicol..

[31] Garg SS, Gupta J, Sharma S, Sahu D (2020). An insight into the therapeutic applications of coumarin compounds and their mechanisms of action. Eur. J. Pharm. Sci..

[32] El Sayed IE-T, Ullah S, Al-Hartomy OA, Hasanein AM, Ahmed AA, Kahilo KA, El-Naggar ME (2022). Synthesis, nanoformulations, and in vitro anticancer activity of N-substituted side chain neocryptolepine scaffolds. Molecules.

[33] Hamulakova S, Janovec L, Soukup O, Jun D, Kuca K (2017). Synthesis, in vitro acetylcholinesterase inhibitory activity and molecular docking of new acridine-coumarin hybrids. Int. J. Biol. Macromol..

[34] Taheri S, Nazifi M, Mansourian M, Hosseinzadeh L, Shokoohinia Y (2019). Ugi efficient synthesis, biological evaluation and molecular docking of coumarin-quinoline hybrids as apoptotic agents through mitochondria-related pathways. Bioorg. Chem..

[35] Fan YL, Ke X, Liu M (2018). Coumarin–triazole hybrids and their biological activities. J. Heterocyclic Chem..

[36] Hueso-Falcón I, Amesty A, Anaissi-Afonso L, Lorenzo-Castrillejo I, Machin F, Estévez-Braun A (2017). Synthesis and biological evaluation of naphthoquinone-coumarin conjugates as topoisomerase II inhibitors. Bioorg. Med. Chem. Lett..

[37] Wang Y, Zhang W, Dong J, Gao J (2020). Design, synthesis and bioactivity evaluation of coumarin-chalcone hybrids as potential anticancer agents. Bioorg. Chem..

[38] Kamath PR, Sunil D, Ajees AA, Pai K, Das S (2015). Some new indole–coumarin hybrids; Synthesis, anticancer and Bcl-2 docking studies. Bioorg. Chem..

[39] Ismail, N. A., Salman, A. A., Yusof, M. S., Soh, S. K., Ali, H. M., & Sarip, R. (2018). The synthesis of a novel anticancer compound, N-(3, 5 Dimethoxyphenyl) acridin-9-amine and evaluation of its toxicity. *Open Chem. J. 5*(1).

[40] El-Bahnsawye M, Hussein MKA, Elmongy EI, Awad HM, Tolan AAE-K, Moemen YS, El-Shaarawy A, El-Sayed IE-T (2022). Design, synthesis, and antiproliferative activity of novel neocryptolepine-rhodanine hybrids. Molecules.

[41] Elebiju OF, Ajani OO, Oduselu GO, Ogunnupebi TA, Adebiyi E (2023). Recent advances in functionalized quinoline scaffolds and hybrids—Exceptional pharmacophore in therapeutic medicine. Front. Chem..

[42] Adhikari S, Mitra AK (2023). Perspective on acridine: A versatile heterocyclic biologically imperative framework. J. Iran. Chem. Soc..

[43] Wang N, Świtalska M, Wang L, Shaban E, Hossain MI, El Sayed IET, Wietrzyk J, Inokuchi T (2019). Structural modifications of nature-inspired indoloquinolines: A mini review of their potential antiproliferative activity. Molecules.

[44] Laponogov I, Sohi MK, Veselkov DA, Pan X-S, Sawhney R, Thompson AW, McAuley KE, Fisher LM, Sanderson MR (2009). Structural insight into the quinolone–DNA cleavage complex of type IIA topoisomerases. Nat. Struct. Mol. Biol..

[45] Koch A, Tamez P, Pezzuto J, Soejarto D (2005). Evaluation of plants used for antimalarial treatment by the Maasai of Kenya. J. Ethnopharmacol..

[46] Utreja D, Jain N, Sharma S (2018). Advances in synthesis and potentially bioactive of coumarin derivatives. Curr. Org. Chem..

[47] Le, T.D., N.N. Pham, & T.C. Nguyen, Preparation and antibacterial activity of some new 4-(2-heterylidenehydrazinyl)-7-chloroquinoline derivatives. *J. Chem.* (2018).

[48] Hassan KM, Shaban E, Elhaddad GM, Shokair SH, Pannipara M, ElSayed IE (2022). Synthesis, printing applications and electrochemical removal of CQAPDN disperse dye incorporating quinoline moiety. J. King Saud Univ. Sci..

[49] Marquez VE, Cranston JW, Ruddon RW, Burckhalter JH (1974). Binding to deoxyribonucleic acid and inhibition of ribonucleic acid polymerase by analogs of chloroquine. J. Med. Chem..

[50] Abd Eldaim MA, Tousson E, El Sayed IET, Abd Elmaksoud AZ, Ahmed AA (2021). Ameliorative effects of 9-diaminoacridine derivative against Ehrlich ascites carcinoma–induced hepatorenal injury in mice. Environ. Sci. Pollut. Res..

[51] Ahmed AA, Awad HM, El-Sayed IE-T, El Gokha AA (2020). Synthesis and antiproliferative activity of new hybrids bearing neocryptolepine, acridine and α-aminophosphonate scaffolds. J. Iran. Chem. Soc..

[52] Sebeka AAH, Osman AM, El Sayed IE-T, El Bahanasawy M, Tantawy MA (2017). Synthesis and antiproliferative activity of novel neocryptolepine-hydrazides hybrids. J. Appl. Pharm. Sci..

[53] Gomaa MS, Ali IA, El Enany G, El Ashry ESH, El Rayes SM, Fathalla W, Ahmed AH, Abubshait SA, Abubshait HA, Nafie MS (2022). Facile synthesis of some coumarin derivatives and their cytotoxicity through VEGFR2 and topoisomerase II inhibition. Molecules.

[54] Liang X, Wu Q, Luan S, Yin Z, He C, Yin L, Zou Y, Yuan Z, Li L, Song X (2019). A comprehensive review of topoisomerase inhibitors as anticancer agents in the past decade. Eur. J. Med. Chem..

[55] Mehndiratta S, Sharma S, Kumar S, Nepali K (2015). Molecular hybrids with anticancer activity. Top. Anti-Cancer Res..

[56] Feng LS, Xu Z, Chang L, Li C, Yan XF, Gao C, Ding C, Zhao F, Shi F, Wu X (2020). Hybrid molecules with potential in vitro antiplasmodial and in vivo antimalarial activity against drug-resistant *Plasmodium*
*falciparum*. Med. Res. Rev..

[57] Paul K, Bindal S, Luxami V (2013). Synthesis of new conjugated coumarin–benzimidazole hybrids and their anticancer activity. Bioorg. Med. Chem. Lett..

[58] Chen H, Li S, Yao Y, Zhou L, Zhao J, Gu Y, Wang K, Li X (2013). Design, synthesis, and anti-tumor activities of novel triphenylethylene–coumarin hybrids, and their interactions with Ct-DNA. Bioorg. Med. Chem. Lett..

[59] Musa MA, Badisa VL, Latinwo LM, Patterson TA, Owens MA (2012). Coumarin-basedbenzopyranone derivatives induced apoptosis in human lung (A549) cancer cells. Anticancer Res..

[60] Avin BV, Thirusangu P, Ranganatha VL, Firdouse A, Prabhakar B, Khanum SA (2014). Synthesis and tumor inhibitory activity of novel coumarin analogs targeting angiogenesis and apoptosis. Eur. J. Med. Chem..

[61] Nofal AE, Elmongy EI, Hassan EA, Tousson E, Ahmed AA, El Sayed IET, Binsuwaidan R, Sakr M (2023). Impact of synthesized indoloquinoline analog to isolates from cryptolepis sanguinolenta on tumor growth inhibition and hepatotoxicity in ehrlich solid tumor-bearing female mice. Cells.

[62] DeLano WL (2002). Pymol: An open-source molecular graphics tool. CCP Newsl. Protein Crystallogr..

[63] Elmongy EI, Altwaijry N, Attallah NG, AlKahtani MM, Henidi HA (2022). In-silico screening of novel synthesized thienopyrimidines targeting fms related receptor tyrosine kinase-3 and their in-vitro biological evaluation. Pharmaceuticals.

