# Supplementary material for: Synthesis and in-vitro anti-proliferative with antimicrobial activity of new coumarin containing heterocycles hybrids
Source: Sci Rep. 2023 Dec 21;13:22791. doi: 10.1038/s41598-023-50170-9 (PMC10733349; doi:10.1038/s41598-023-50170-9)
Supplement: Supplementary file 1 — Supplementary Information. [file 41598_2023_50170_MOESM1_ESM.docx]

**Synthesis and *in-vitro* anti-proliferative with antimicrobial activity of new coumarin containing heterocycles hybrids**

| **3** |   Analysis of the GC/MS revealed MS peaks at m/z 190, 173,164  H. Ç., Hasniye, Y., & Oktay, S. (2019). Comparison of Antioxidant Activities of Mono-, Di-and Tri-substituted Coumarins. *Journal of the Turkish Chemical Society Section A: Chemistry*, *7*(1), 87-96 |
| --- | --- |
| **6a** |  |
|  |  |
|  | **Figure S1.** Mass spectra of 7-chloro-4-hydrazinylquinoline **(6a)**. **EI-MS**, m ̸z (C_9_H_8_ClN_3_) calcd, 193.04; found, 192.0 [M-1]^+^=100%, 194.0 [M+1] ^+^ . |
| **6b** |  |
|  |  |
|  | **Figure S2.** Mass spectra of  *N^1^*-(7-chloroquinolin-4-yl)ethane-1,2-diamin **(6b)**. **EI-MS**, m ̸z (C_11_H_12_ClN_3_) calcd, 221.69; found, 221.1 [M^+^] = 12.3% , 223.8 [M+2] ^+^ . |
| **6c** |  |
|  | 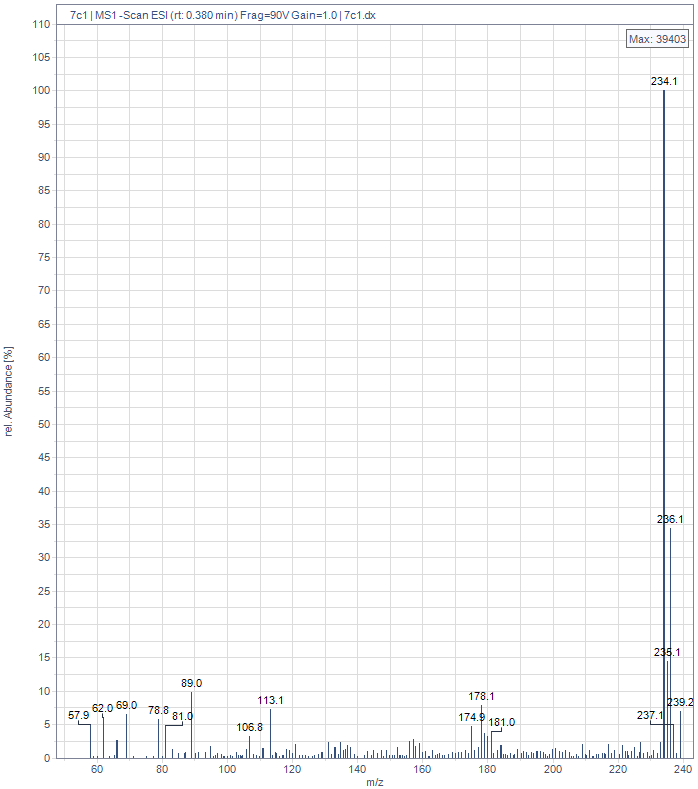 |
|  | **Figure S3.** Mass spectra of *N^1^*-(7-chloroquinolin-4-yl)propane-1,3-diamine **(6c)**. **EI-MS**, m ̸z (C_12_H_14_ClN_3_) calcd, 235.09; found, 234.1 [M-1]^+^ =100%, 236.1[M+1] ^+^ . |
| **6d** |  |
|  | 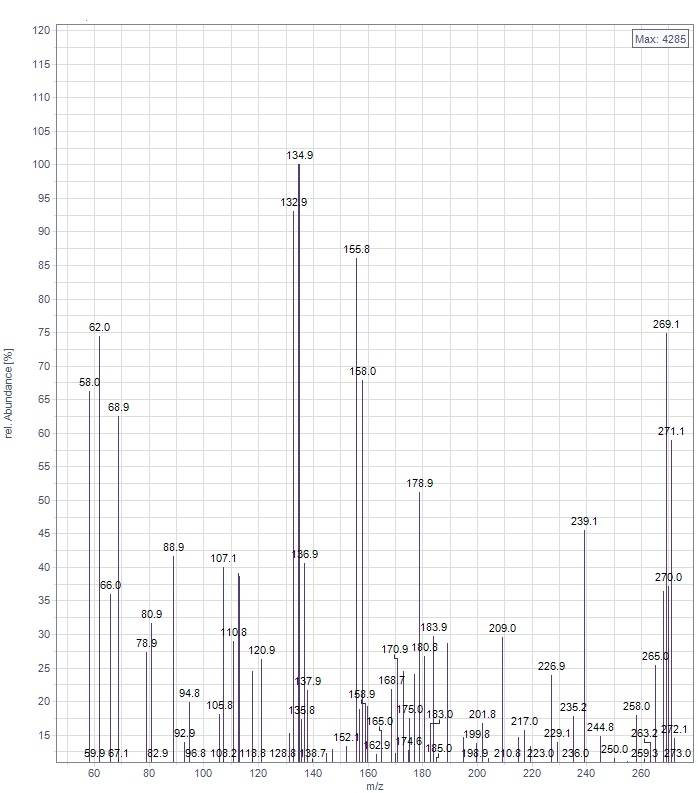 |
|  | **Figure S4.** Mass spectra of *N^1^*-(7-chloroquinolin-4-yl)benzene-1,4-diamine **(6d)**.**EI-MS**, m ̸z (C_15_H_12_ClN_3_) calcd, 269.07; found, 269.1 [M^+^] =75%, 271.1[M+2] ^+^ . |
| **6e** |   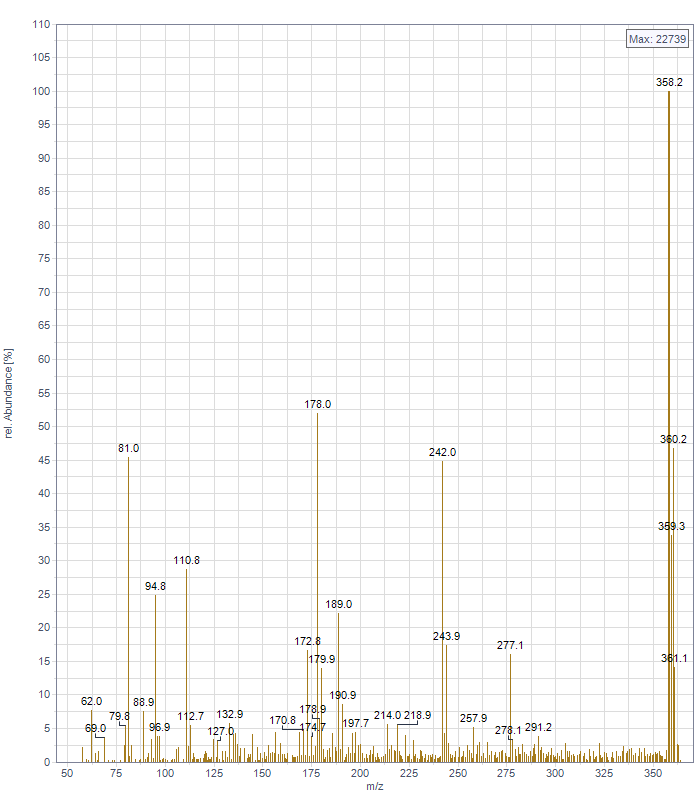  **Figure S5** Mass spectra of *N*-(4-(4-aminobenzyl)phenyl)-7-chloroquinolin-4-amine **(6e)**. **EI-MS**, m ̸z (C_22_H_18_ClN_3_) calcd, 359.12; found, 358.2 [M-1]^+^ =100%, 360.2[M+1] ^+^ . |
|  | **7a**  |
|  |  |
|  |  |
| **c** | **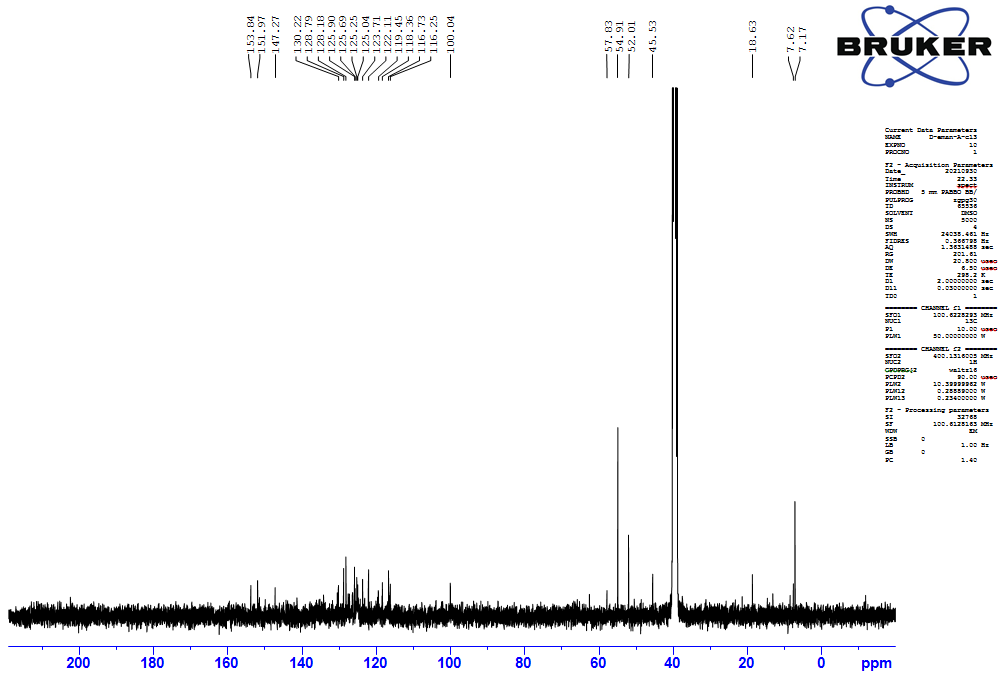** |
| **d** | **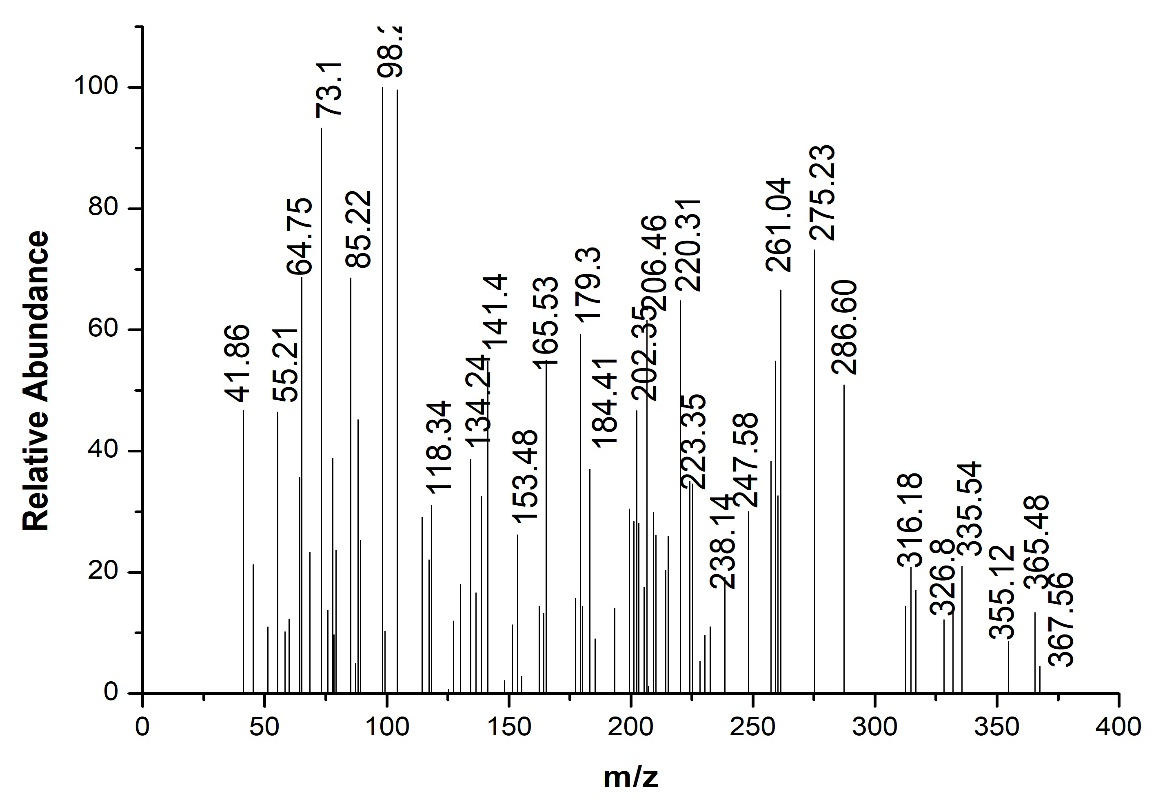** |
|  | **Figure S6.** FTIR (a), ^1^H-NMR (b), ^13^C-NMR (C), Mass spectra (d) of *N'*-(7-chloroquinolin-4-yl)-2-oxo-2*H*-chromene-3-carbohydrazide **(7a)**. |
| **7b** |  |
| **a** | **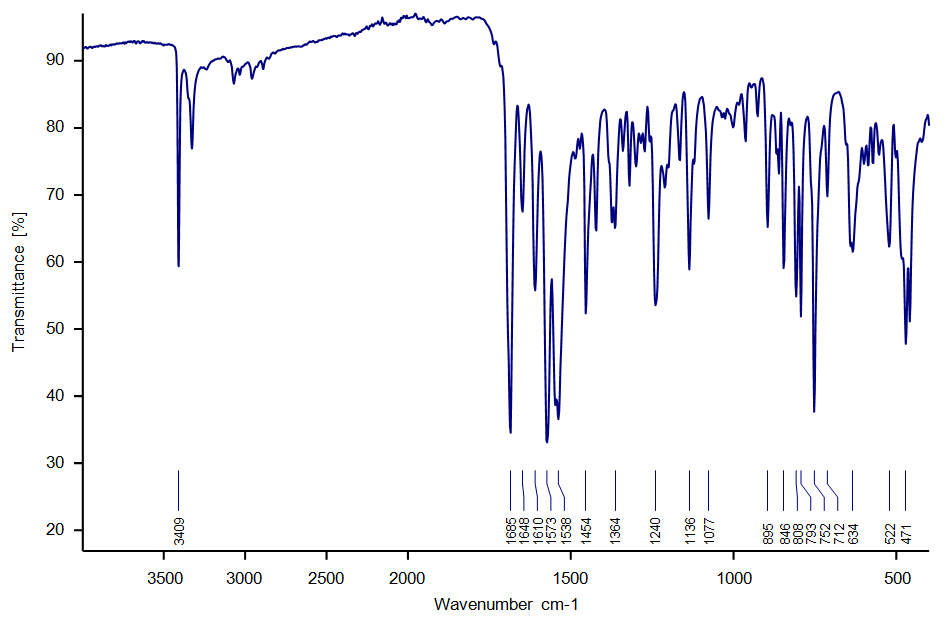** |
| **b** | **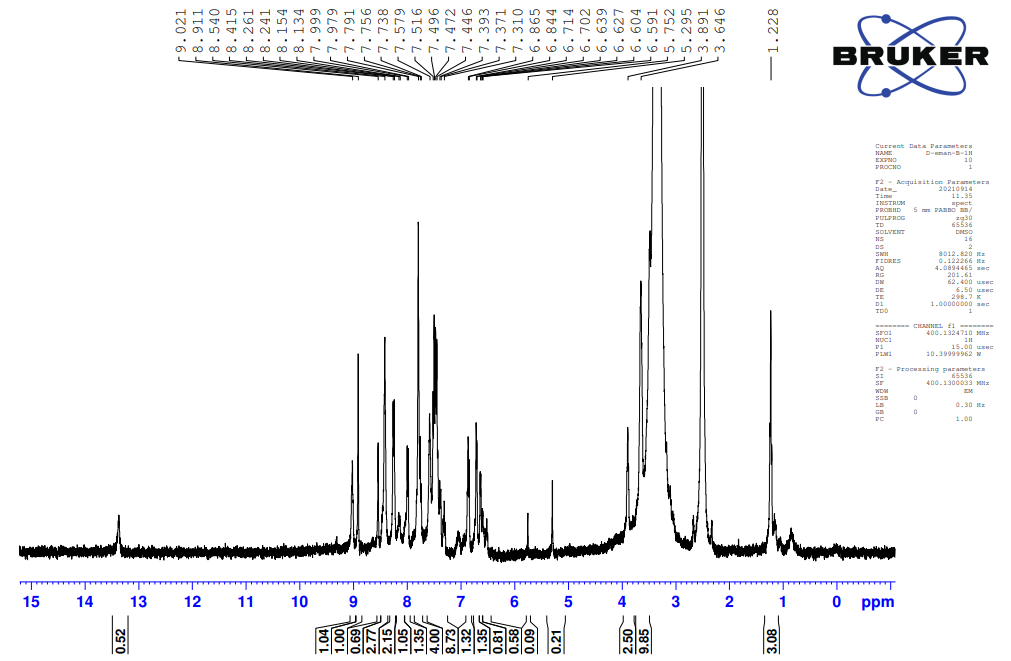** |
| **c** | **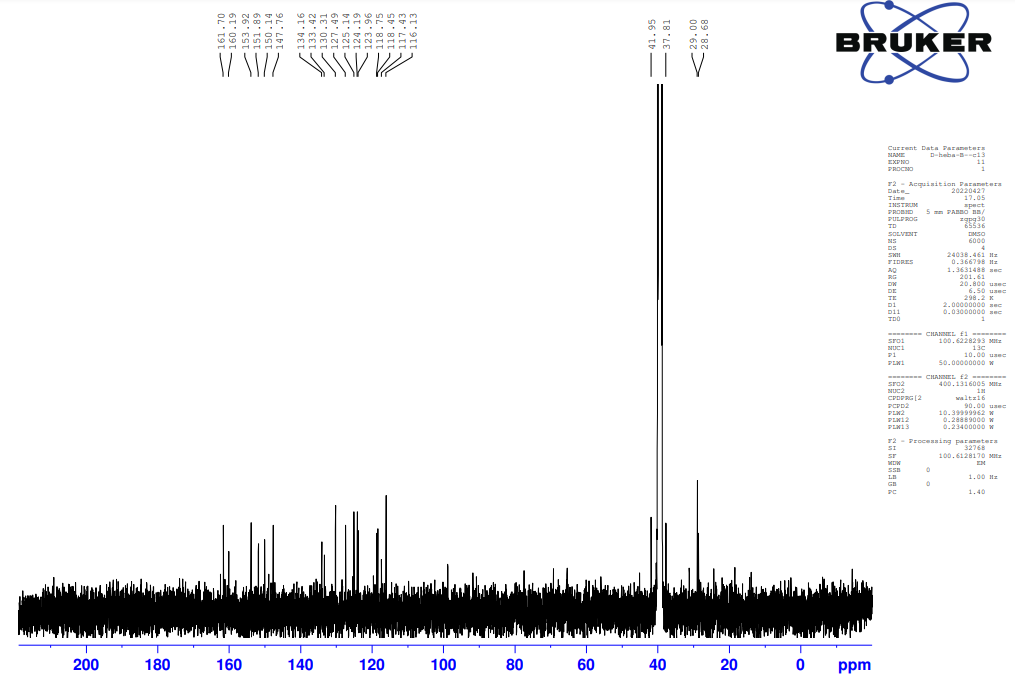** |
| **d** | **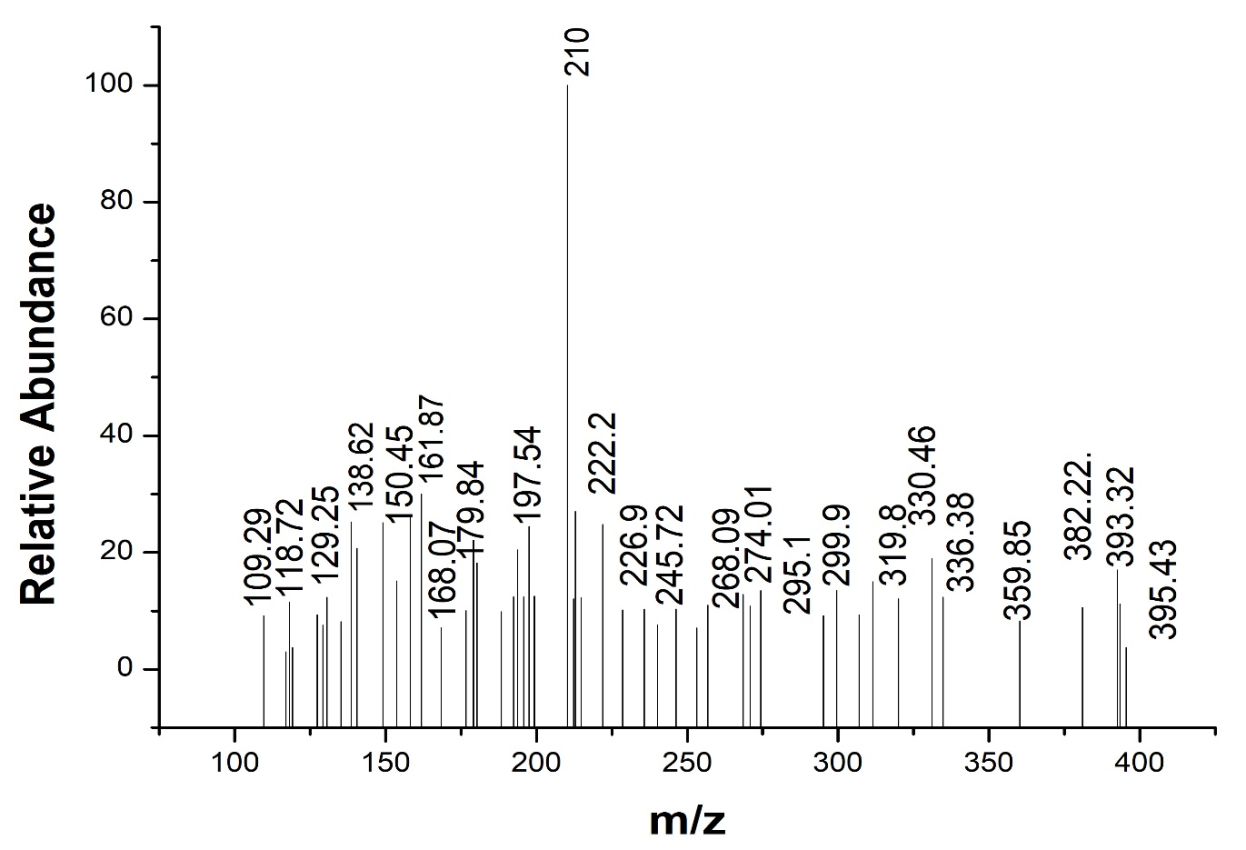** |
|  | **Figure S7.** FTIR (a), ^1^H-NMR (b), ^13^C-NMR (C), Mass spectra (d) of *N*-(2-((7-chloroquinolin-4-yl)amino)ethyl)-2-oxo-2*H*-chromene-3-carboxamide) **(7b)**. |
| **7c** |  |
| **a** | **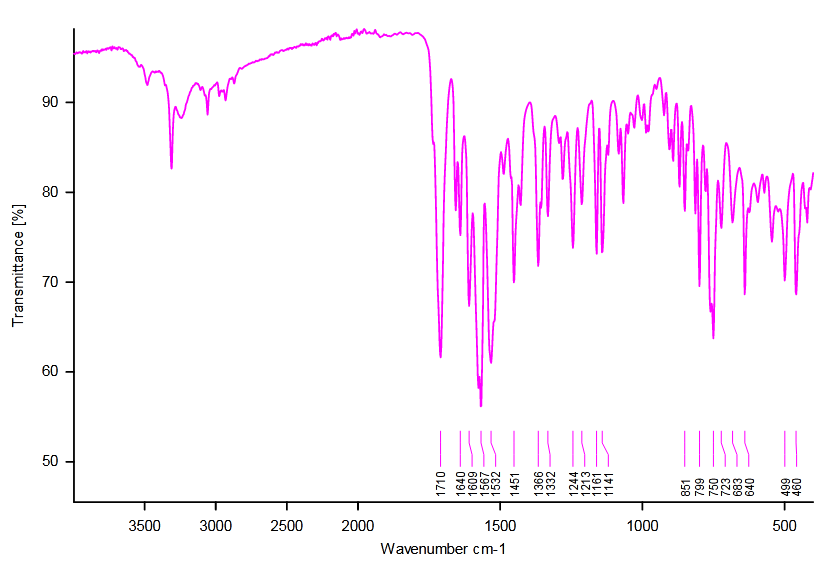** |
| **b** | **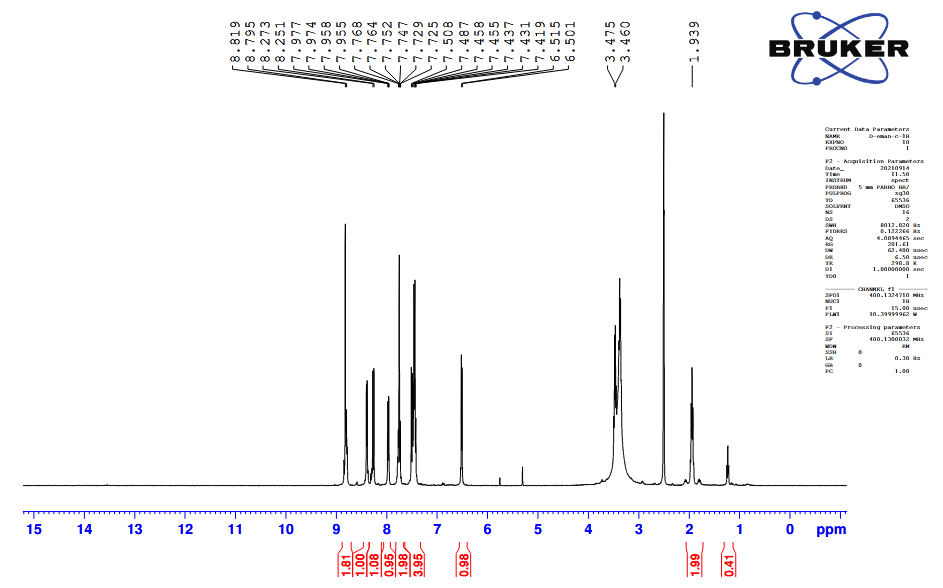** |
| **c** | **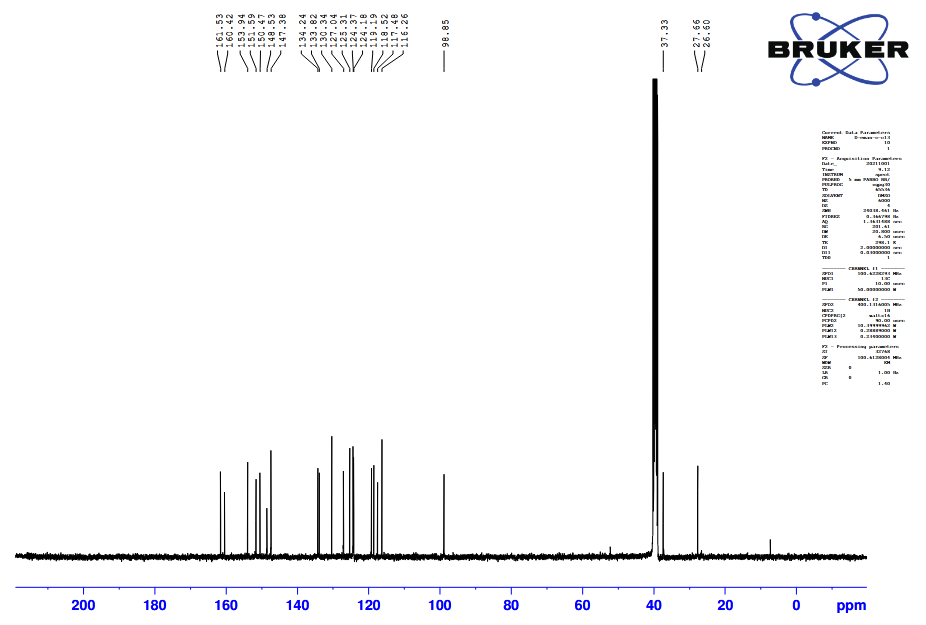** |
| **d** | **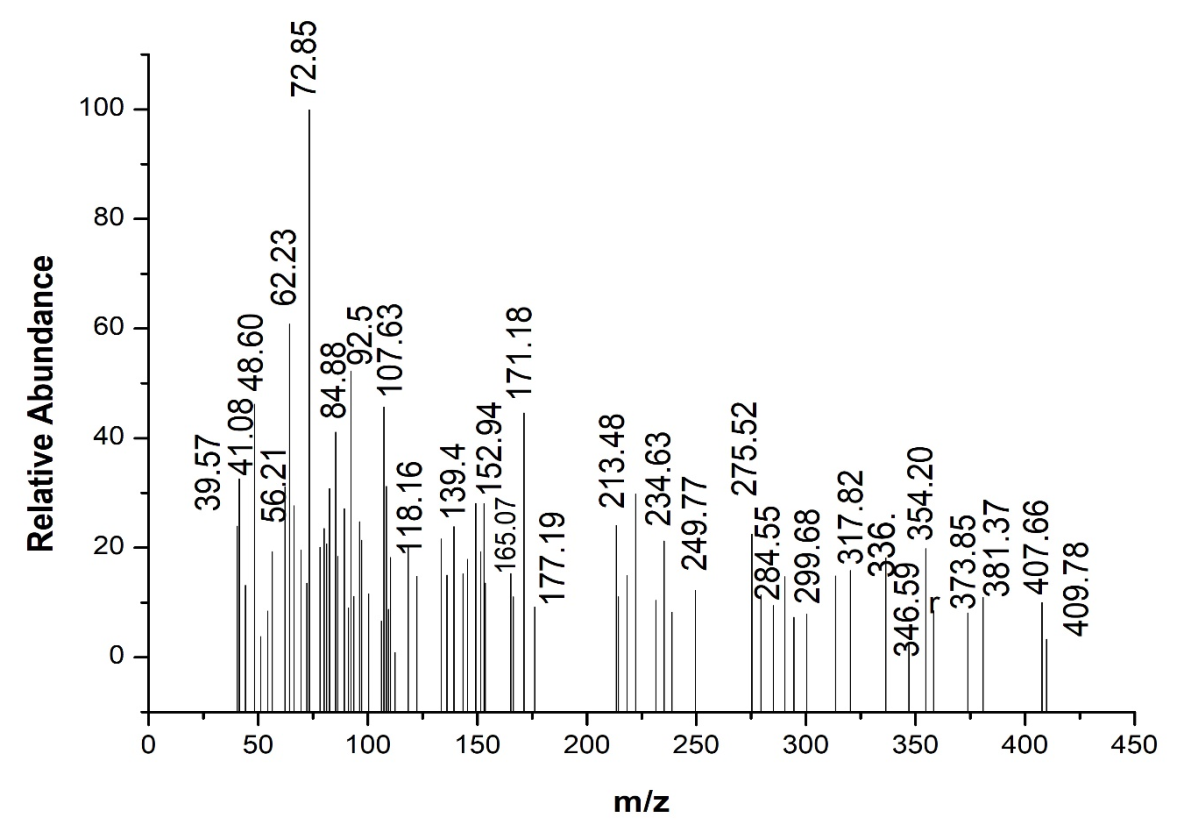** |
|  | **Figure S8.** FTIR (a), ^1^H-NMR (b), ^13^C-NMR (C), Mass spectra (d) of *N*-(3-((7-chloroquinolin-4-yl)amino)propyl)-2-oxo-2*H*-chromene-3-carboxamide **(7c)**. |
| **7d** |  |
| **a** | **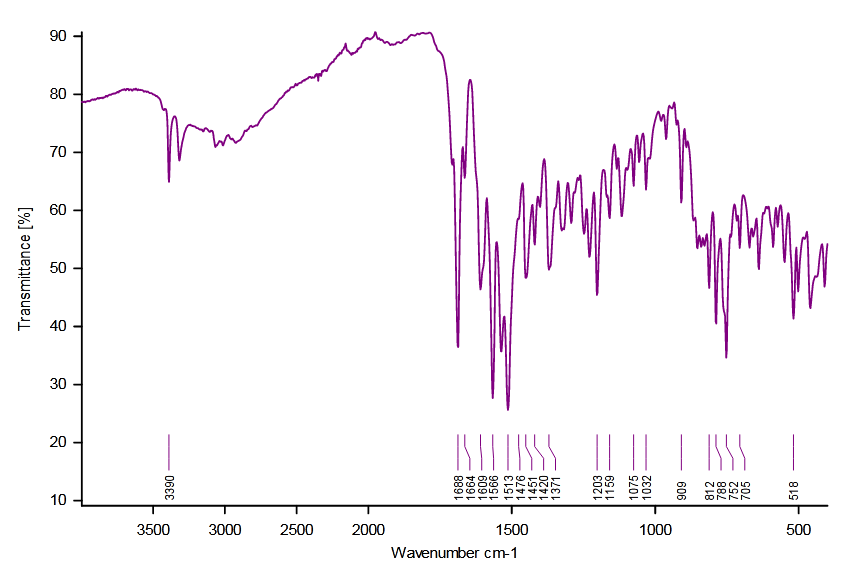** |
| **b** | **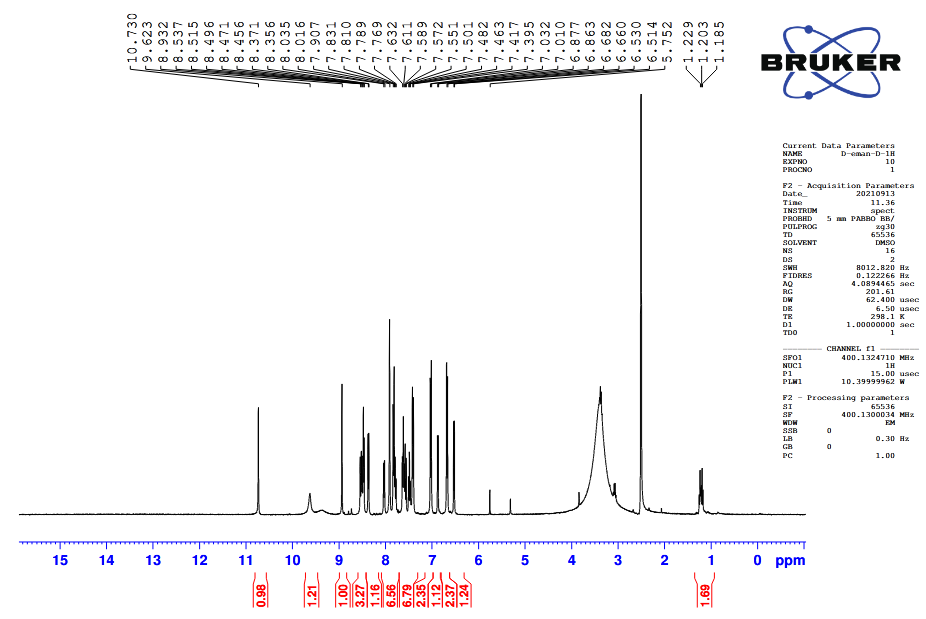** |
| **c** | **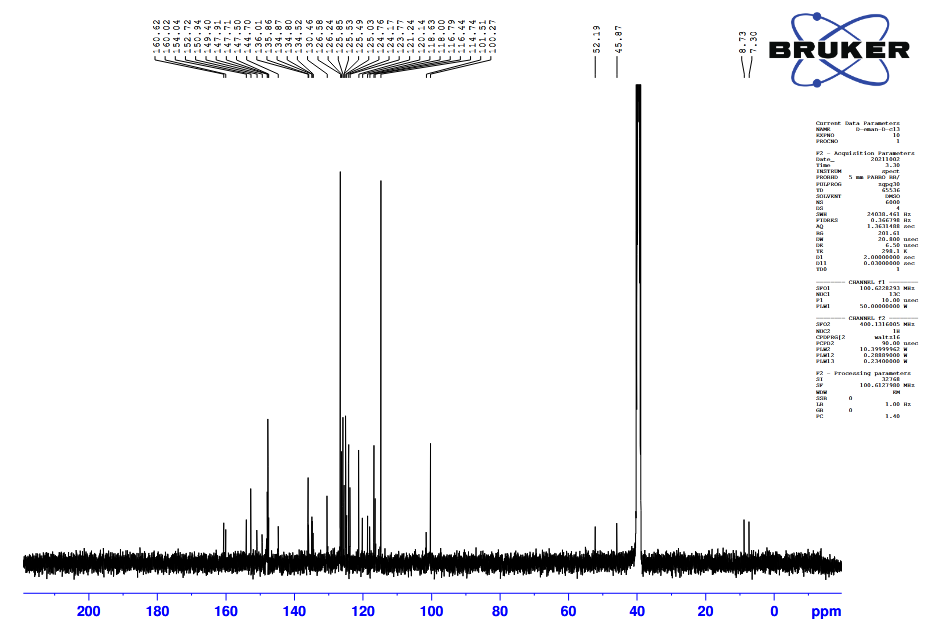** |
| **d** | **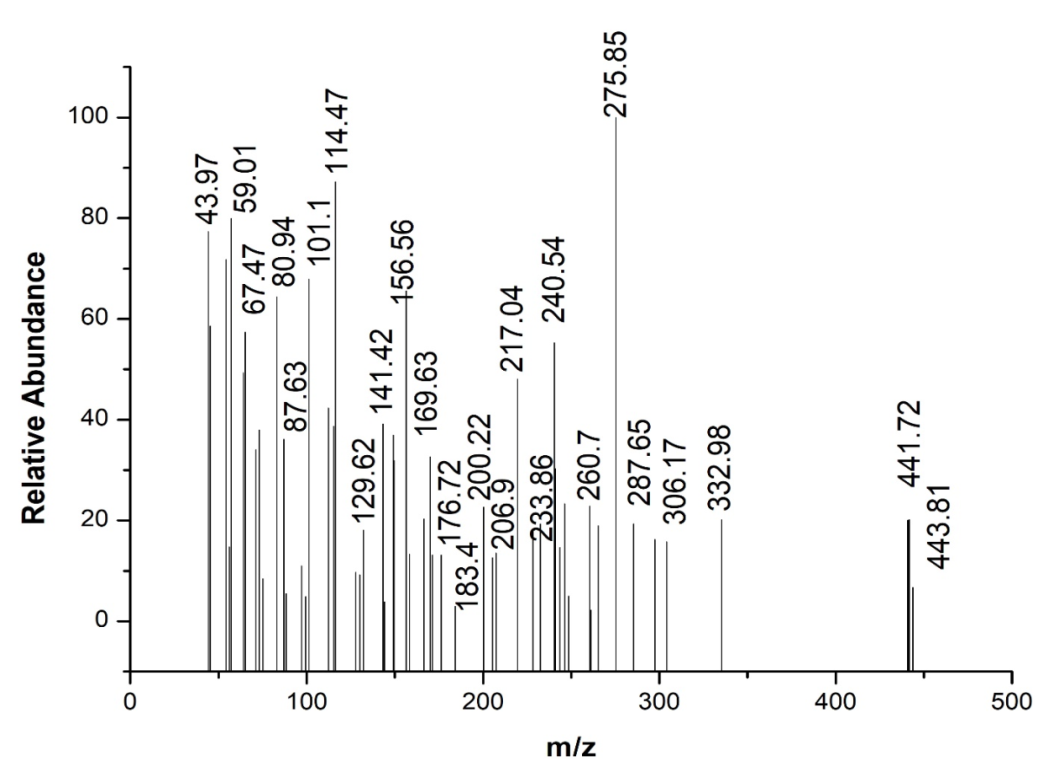** |
|  | **Figure S9.** FTIR (a), ^1^H-NMR (b), ^13^C-NMR (c), Mass spectra (d) of *N*-(4-((7-chloroquinolin-4-yl)amino)phenyl)-2-oxo-2*H*-chromene-3-carboxamide **(7d)**. |
| **7e** |  |
| **a** | **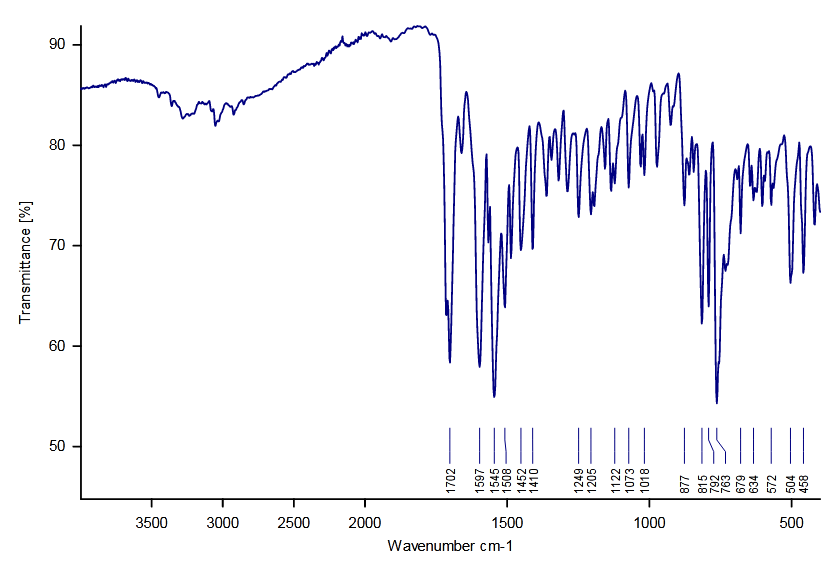** |
| **b** | **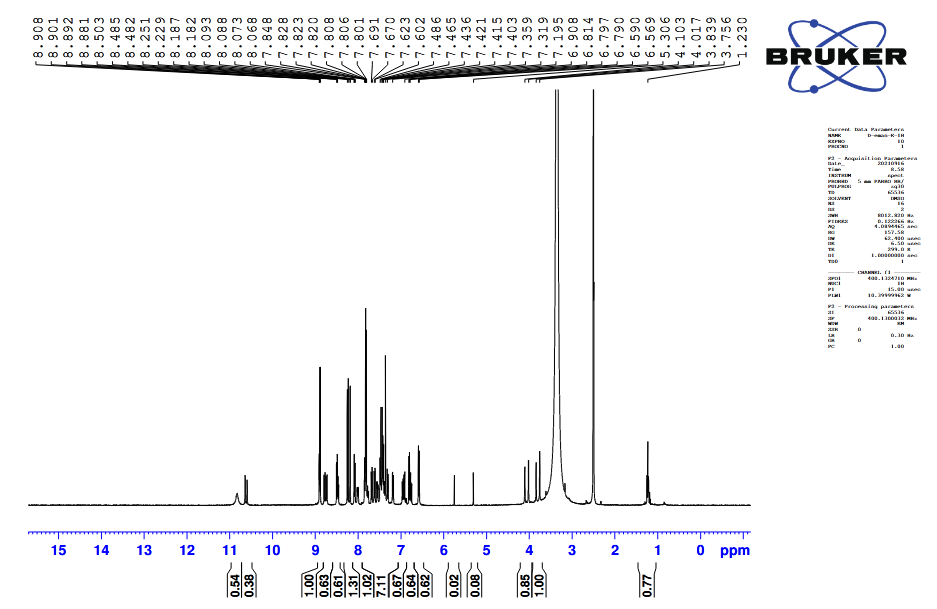** |
| **c** | **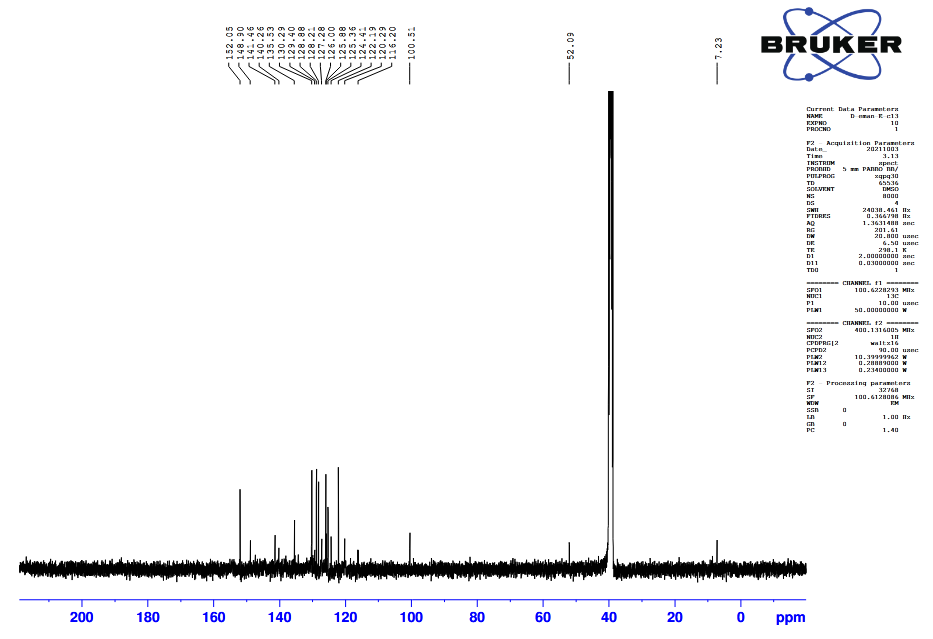** |
| **d** | **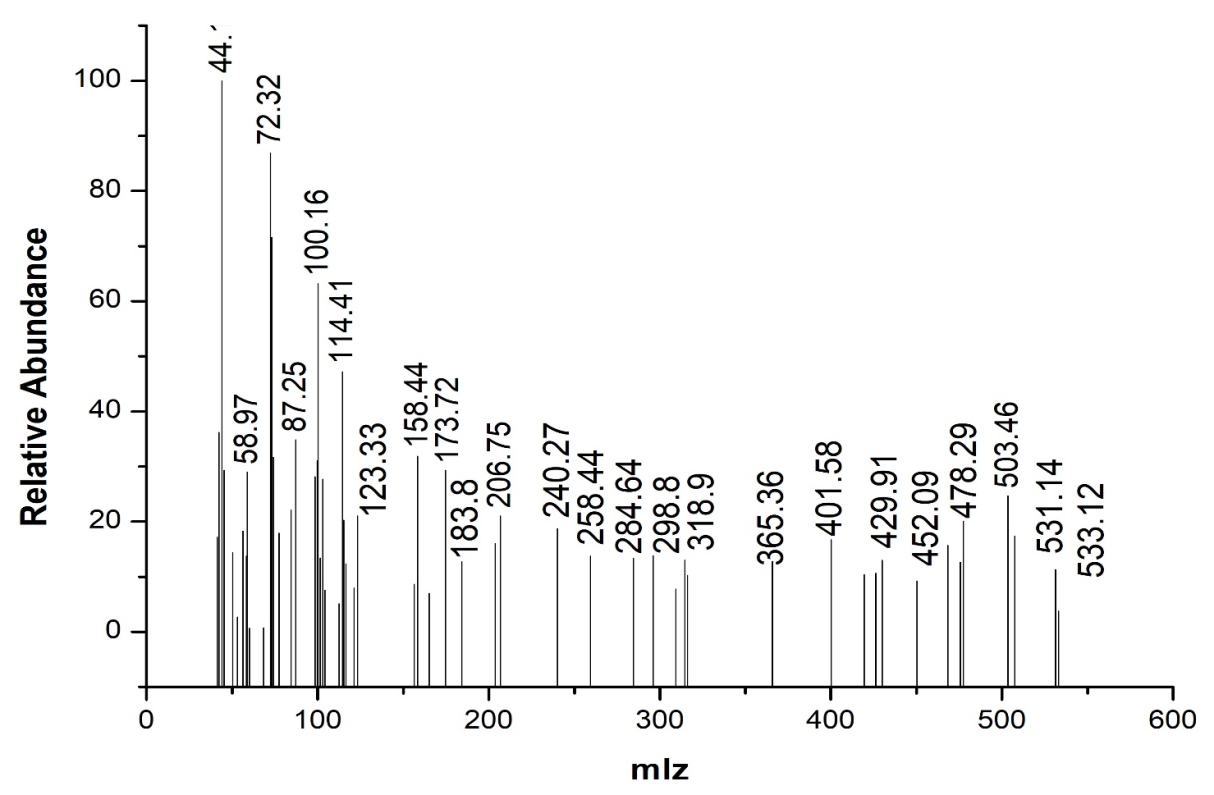** |
|  | **Figure S10.** FTIR (a), ^1^H-NMR (b), ^13^C-NMR (c), Mass spectra (d) of *N*-(4-(4-((7-chloroquinolin-4-yl)amino)benzyl)phenyl)-2-oxo-2*H*-chromene-3-carboxamide **(7e)**. |
| **10b** |  |
| **a** | **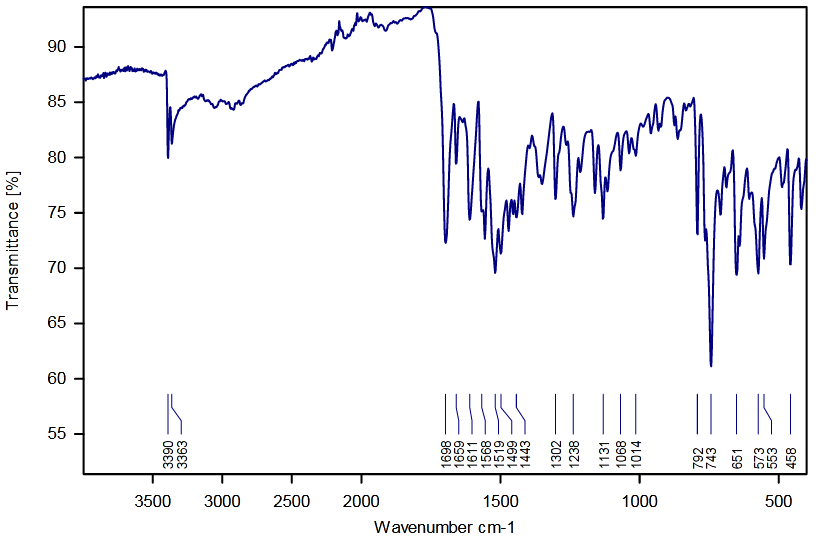** |
| **b** | **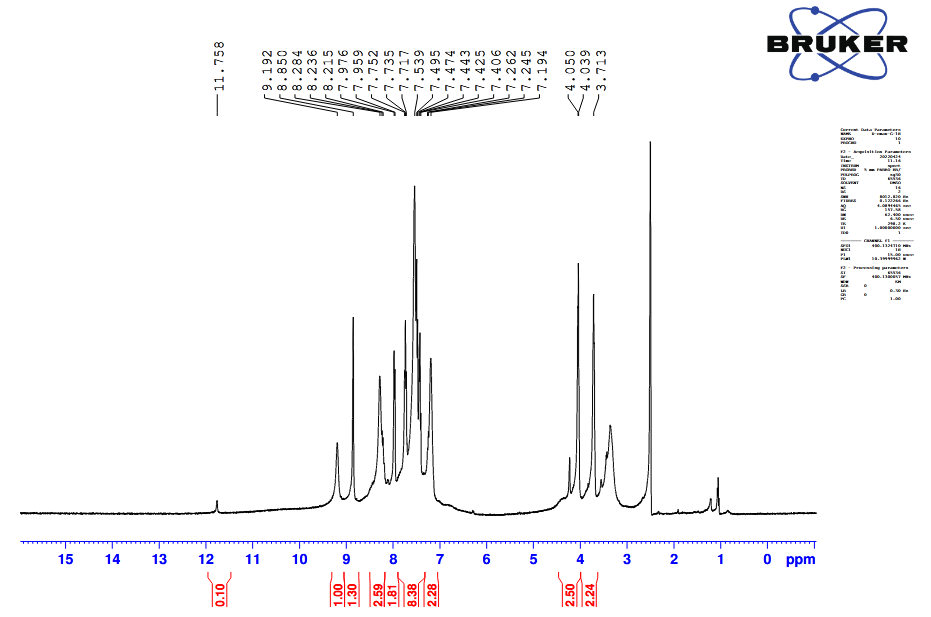** |
| **c** | **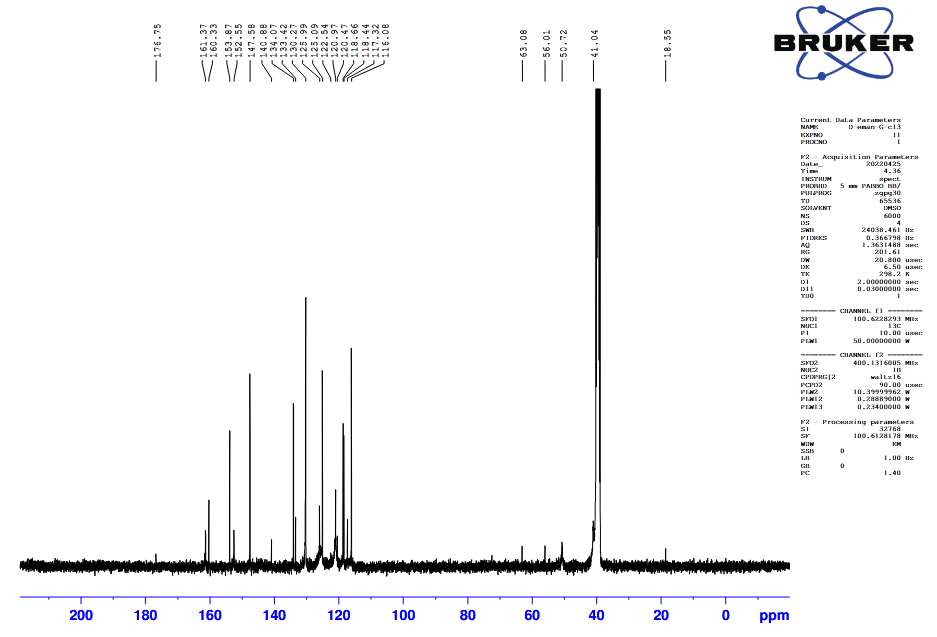** |
| **d** | **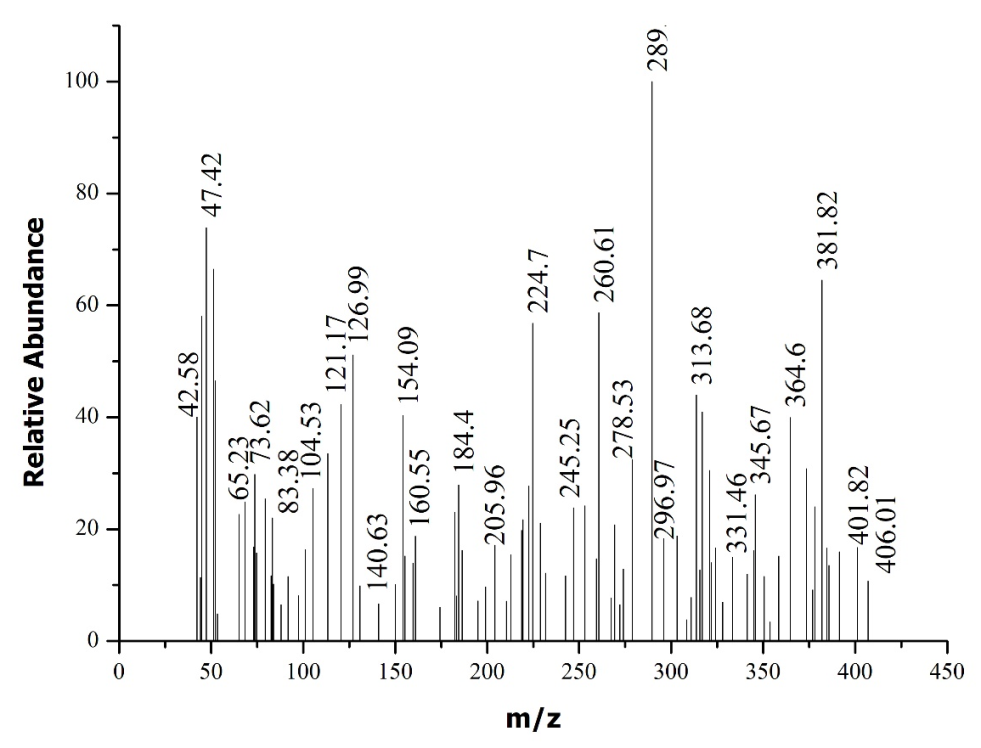** |
|  | **Figure S11.** FTIR (a), ^1^H-NMR (b), ^13^C-NMR (c), Mass spectra (d) of *N*-(2-(acridin-9-ylamino)ethyl)-2-oxo-2*H*-chromene-3-carboxamide **(10b)**. |
| **10c** |  |
| **a** | **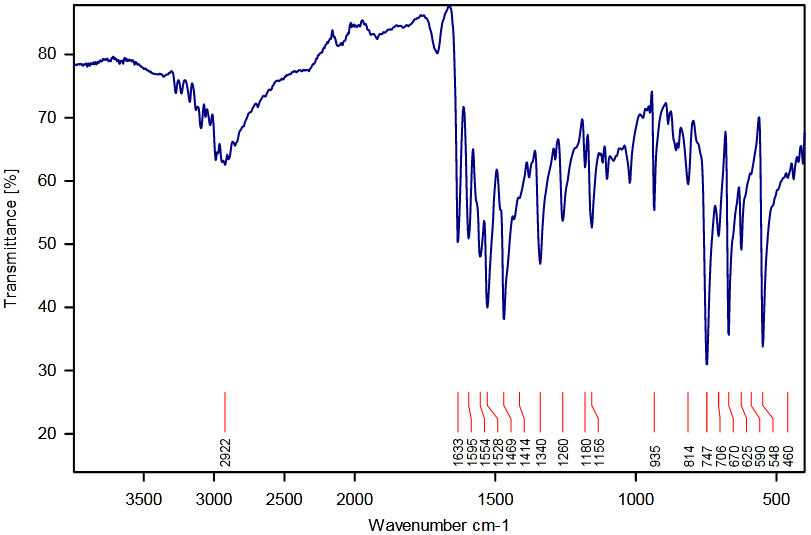** |
| **b** | **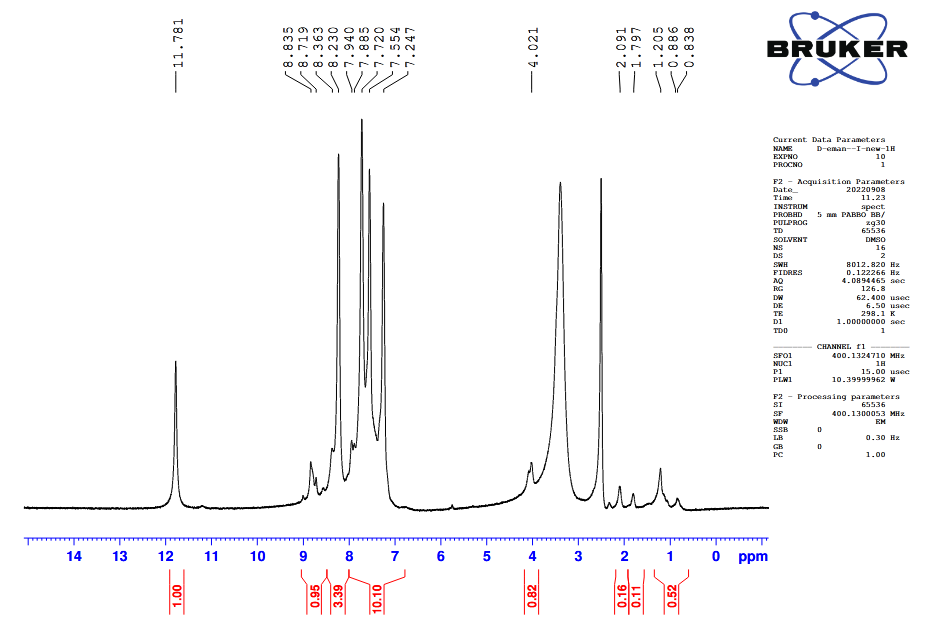** |
| **c** | **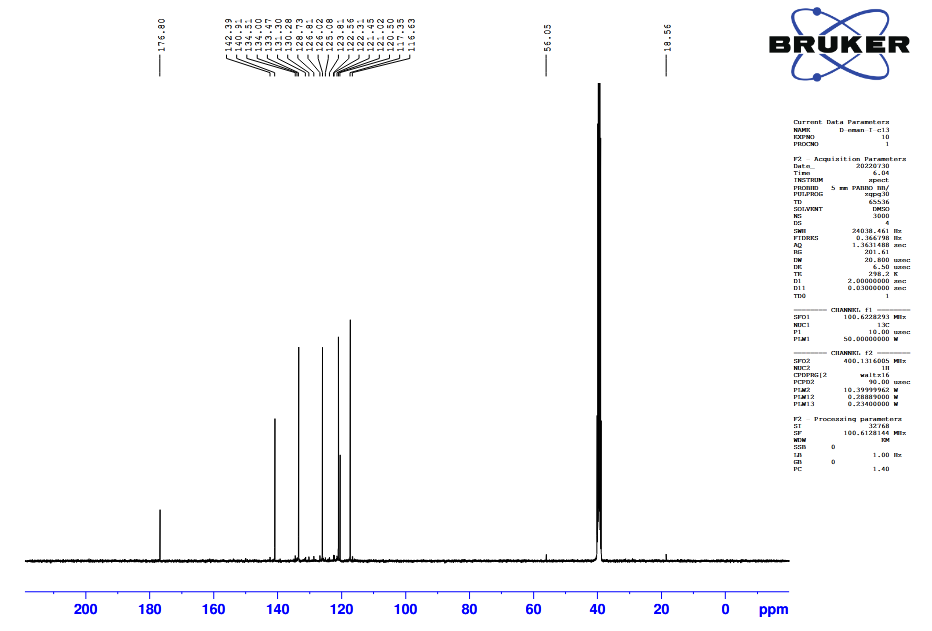** |
| **d** | **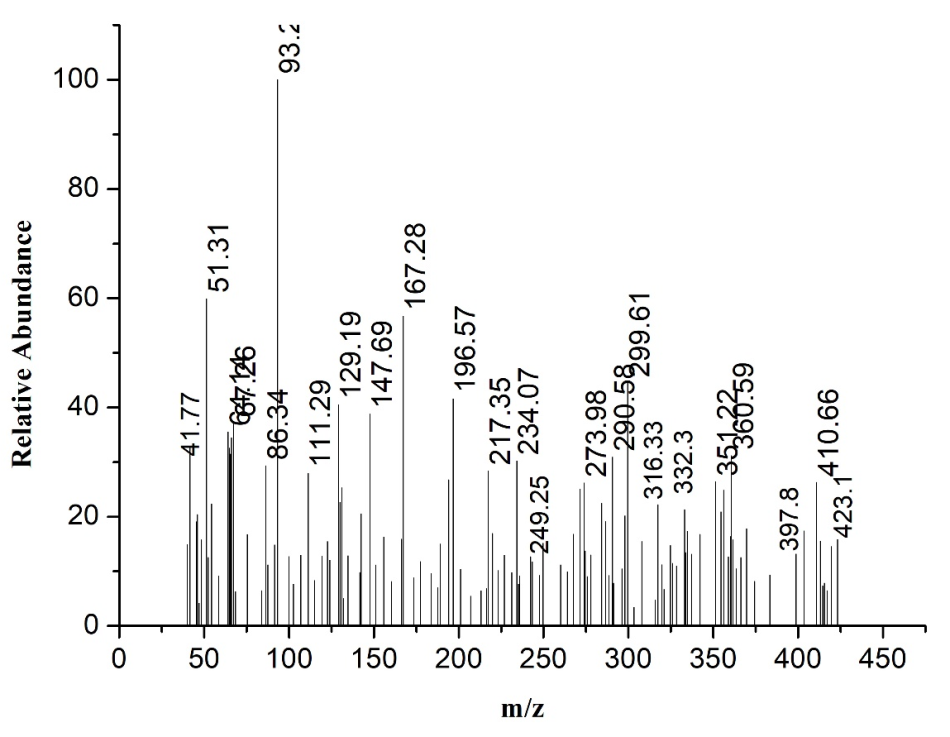** |
|  | **Figure S12**. FTIR (a), ^1^H NMR (b), ^13^C NMR (c), Mass spectra (d) of *N*-(2-(acridin-9-ylamino)propyl)-2-oxo-2*H*-chromene-3-carboxamide **(10c)**. |
| **13b** |  |
| **a** | **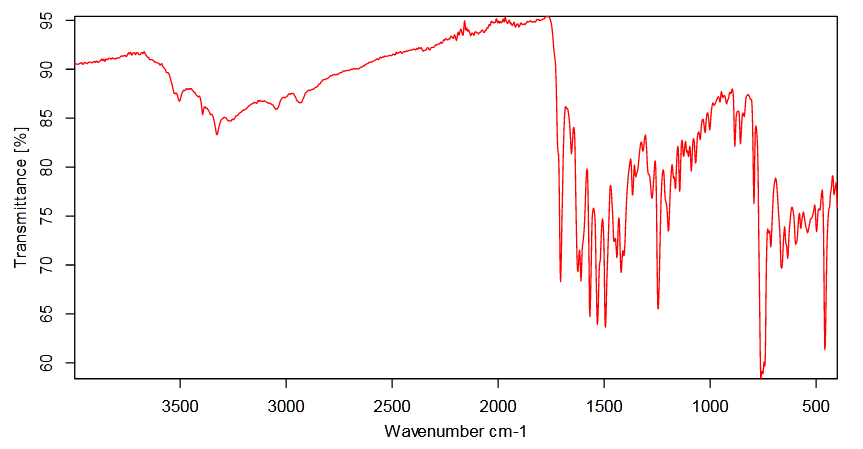** |
| **b** | **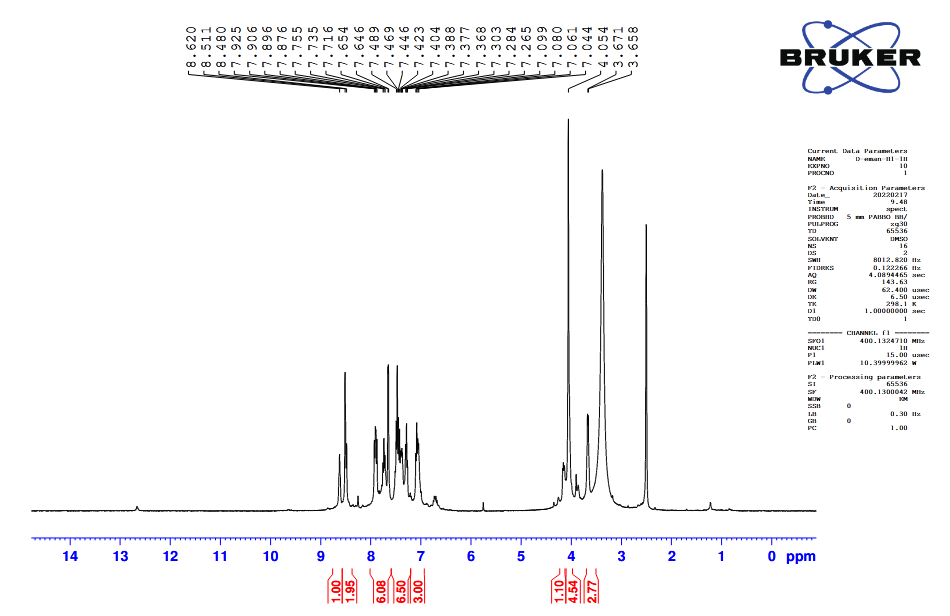** |
| **c** | **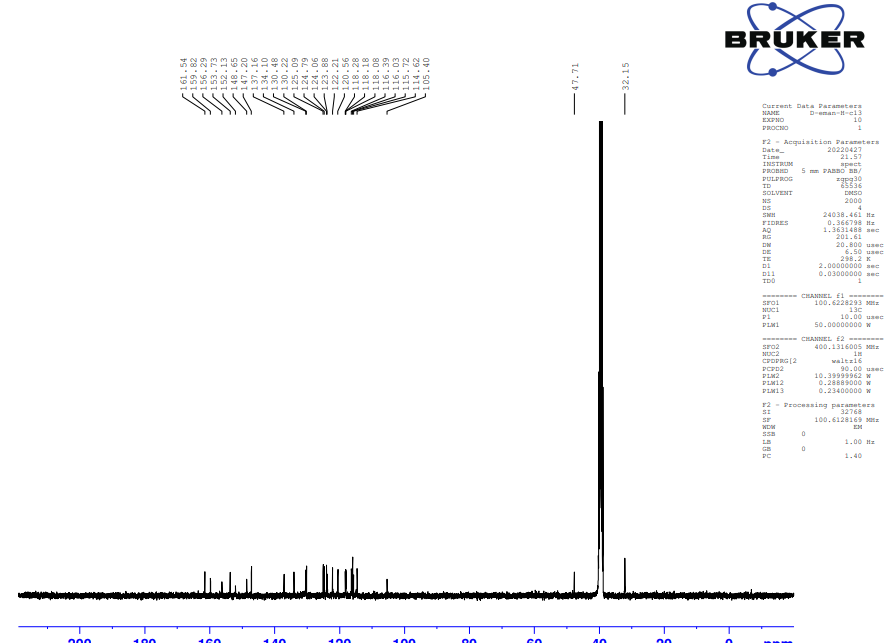** |
| **d** | **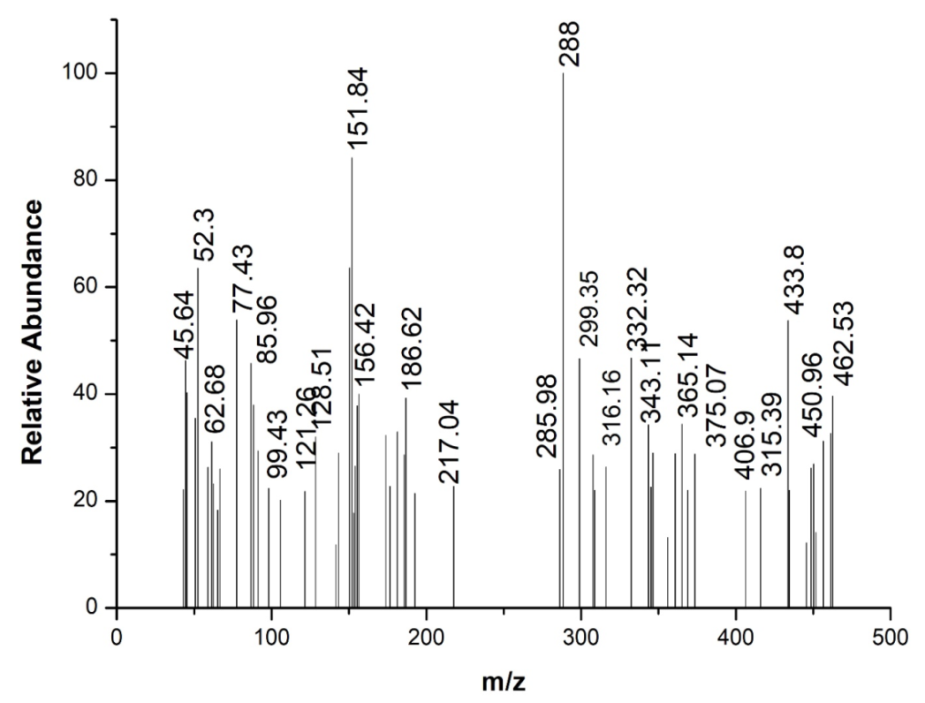** |
|  | **Figure S13.** FT-IR (a), ^1^H-NMR (b), ^13^C-NMR (c), Mass spectra (d) of *N*-(2-((5-methyl-5*H*-indolo[2,3-b]quinolin-11-yl)amino)ethyl)-2-oxo-2*H*-chromene-3-carboxamide **(13b)**. |
| **13c** |  |
| **a** | **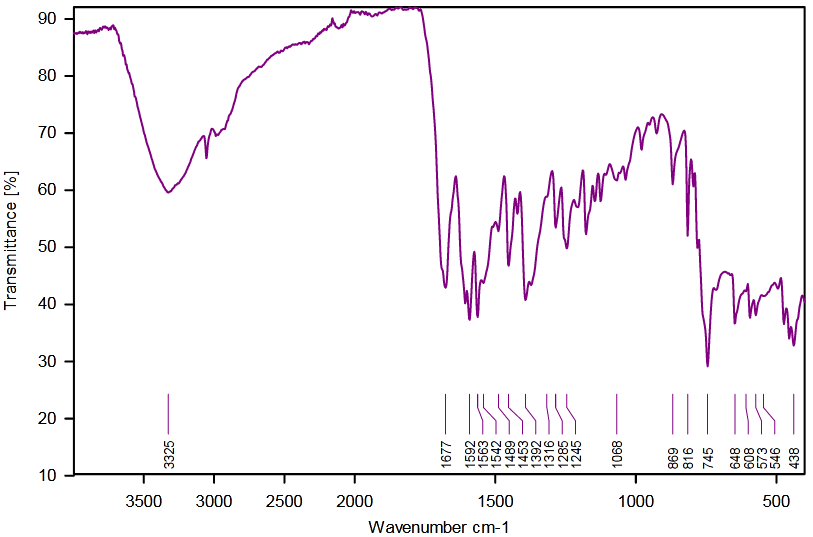** |
| **b** | **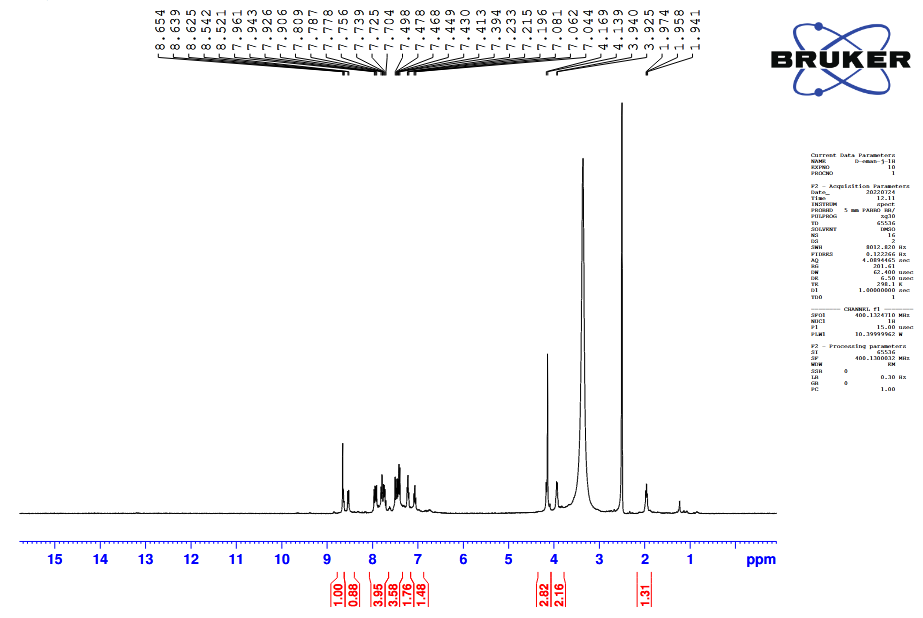** |
| **c** | **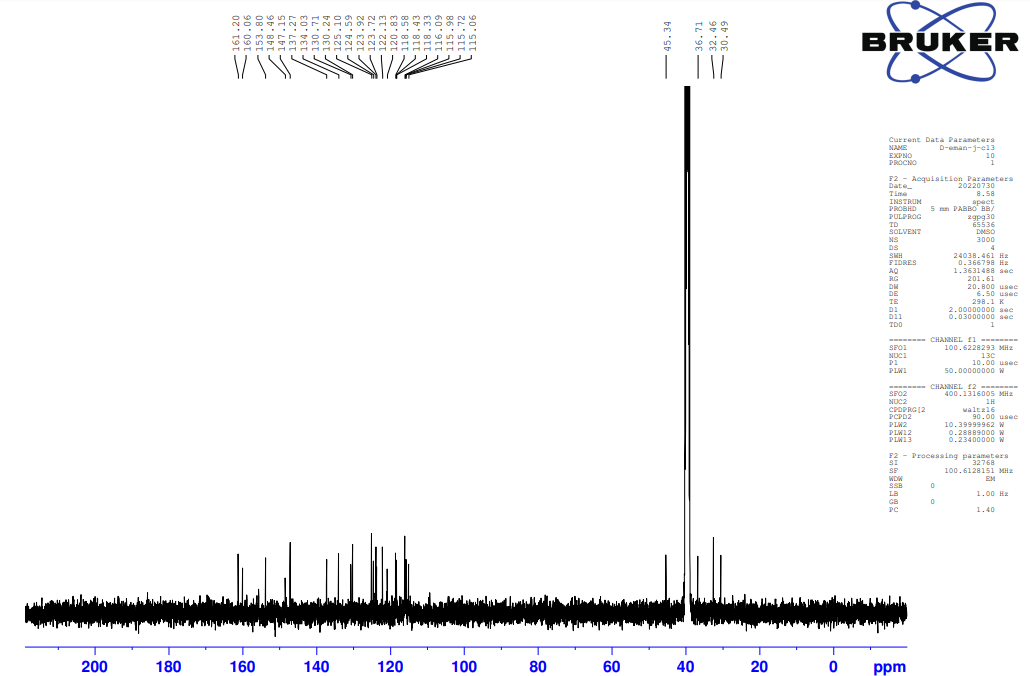** |
| **d** | **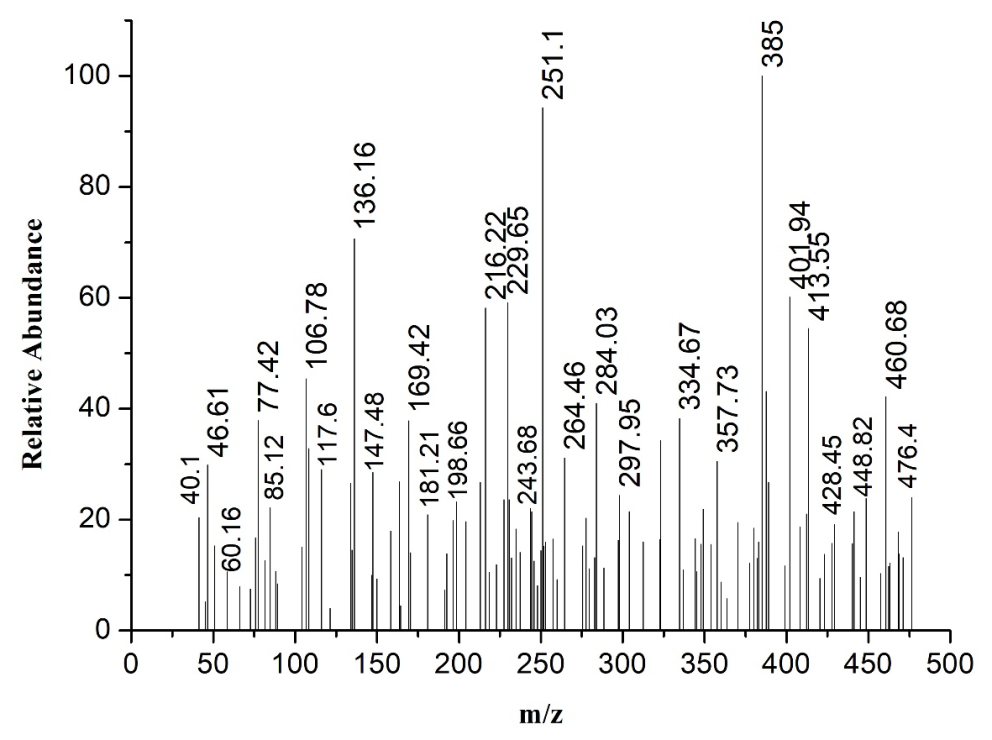** |
|  | **Figure S14.** FTIR (a), ^1^H NMR (b), ^13^C NMR (c), Mass spectra (d) of *N*-(2-((5-methyl-5*H*-indolo[2,3-b]quinolin-11-yl)amino)propyl)-2-oxo-2*H*-chromene-3-carboxamide **(13c)**. |
